# Gingival proteomics reveals the role of TGF beta and YAP/TAZ signaling in Raine syndrome fibrosis

**DOI:** 10.1038/s41598-024-59713-0

**Published:** 2024-04-25

**Authors:** Cláudio Rodrigues Rezende Costa, Rym Chalgoumi, Amina Baker, Clément Guillou, Paulo Marcio Yamaguti, Victor Simancas Escorcia, Lilia Abbad, Bruna Rabelo Amorin, Caroline Lourenço de Lima, Vidjea Cannaya, Mourad Benassarou, Ariane Berdal, Christos Chatziantoniou, Olivier Cases, Pascal Cosette, Renata Kozyraki, Ana Carolina Acevedo

**Affiliations:** 1 Centre de Recherche des Cordeliers, Sorbonne Université, INSERM, Université de Paris Cité, Oral Molecular Pathophysiology, 75006 Paris, France; 2 Oral Center for Inherited Diseases, University Hospital of Brasília, Oral Histopathology Laboratory, Department of Dentistry, Health Sciences Faculty, University of Brasília (UnB), Brasília, Brazil; 3 Department of Dentistry, Health Group of Natal (GSAU-NT), Brazilian Air Force, Natal, Parnamirim, Brazil; 4 Rouen University, INSA Rouen Normandie, CNRS, Normandie Univ, PBS UMR 6270, 76000 Rouen, France; 5 Rouen University, INSERM US51, CNRS UAR 2026, HeRacles PISSARO, 76000 Rouen, France; 6 Grupo de Investigación GENOMA, Universidad del Sinú, Cartagena, Colombia; 7 MRS1155, INSERM, Sorbonne Université, 75020 Paris, France; 8 Service de Chirurgie Maxillo-Faciale et Stomatologie, Hôpital de La Pitié Salpétrière, Sorbonne Université, 75006 Paris, France; 9 CRMR O-RARES, Hôpital Rothshild, UFR d’Odontologie-Garancière, Université de Paris Cité, 75012 Paris, France; 10 Present Address: Rouen University, UFR SANTE ROUEN NORMANDIE, Inserm 1096, 76000 Rouen, France

**Keywords:** FAM20C, FAM20A, Secretome, Proteome, Raine syndrome, TGF beta, YAP/TAZ, Gingival fibroblast, Ectopic mineralization, Gingival fibromatosis, Fibrosis, Collagen, Periostin, Cell adhesion, Cell signalling, Mechanisms of disease, Cell biology, Diseases

## Abstract

Raine syndrome (RNS) is a rare autosomal recessive osteosclerotic dysplasia. RNS is caused by loss-of-function disease-causative variants of the *FAM20C* gene that encodes a kinase that phosphorylates most of the secreted proteins found in the body fluids and extracellular matrix. The most common RNS clinical features are generalized osteosclerosis, facial dysmorphism, intracerebral calcifications and respiratory defects. In non-lethal RNS forms, oral traits include a well-studied hypoplastic amelogenesis imperfecta (AI) and a much less characterized gingival phenotype. We used immunomorphological, biochemical, and siRNA approaches to analyze gingival tissues and primary cultures of gingival fibroblasts of two unrelated, previously reported RNS patients. We showed that fibrosis, pathological gingival calcifications and increased expression of various profibrotic and pro-osteogenic proteins such as POSTN, SPARC and VIM were common findings. Proteomic analysis of differentially expressed proteins demonstrated that proteins involved in extracellular matrix (ECM) regulation and related to the TGFβ/SMAD signaling pathway were increased. Functional analyses confirmed the upregulation of TGFβ/SMAD signaling and subsequently uncovered the involvement of two closely related transcription cofactors important in fibrogenesis, Yes-associated protein (YAP) and transcriptional coactivator with PDZ-binding motif (TAZ). Knocking down of *FAM20C* confirmed the TGFβ-YAP/TAZ interplay indicating that a profibrotic loop enabled gingival fibrosis in RNS patients. In summary, our in vivo and in vitro data provide a detailed description of the RNS gingival phenotype. They show that gingival fibrosis and calcifications are associated with, and most likely caused by excessed ECM production and disorganization. They furthermore uncover the contribution of increased TGFβ–YAP/TAZ signaling in the pathogenesis of the gingival fibrosis.

## Introduction

Family with sequence similarity 20 (FAM20) is a gene family of atypical secreted protein kinases comprising three members in vertebrates, FAM20A, FAM20B and FAM20C. FAM20B, the ancestral template protein for the FAM20 family is a xylosylkinase that phosphorylates xylose residues in the glycosaminoglycan-protein linkage regions of proteoglycan and promotes their production^1^. Mutations in *FAM20B* cause severe neonatal short-limb dysplasia^2^. FAM20A is a pseudokinase; it lacks active site residues and cannot hydrolyze ATP but binds to and allosterically activates FAM20C^3^. Disease-causative variants in *FAM20A* lead to enamel renal syndrome (ERS), a rare hereditary disorder combining AI, gingival fibrosis and nephrocalcinosis^4–10^. FAM20C conserved from nematodes to humans, is the genuine Golgi casein kinase that phosphorylates the S-x-E/pS motif of secreted proteins^11–13^. It is a ubiquitously expressed, 584 amino acids protein. FAM20C contains a catalytic pocket, a FAM20A binding domain and three distinct N-glycosylation sites necessary for protein folding and secretio^14–16^. FAM20C is required for the phosphorylation of ECM proteins involved in the biomineralization of bone and teeth^17–20^. Other FAM20C functions include the regulation of endoplasmic reticulum homeostasis, coagulation and cardiac function^21–26^.

Loss-of-function variants in the *FAM20C* gene result in RNS (OMIM #259775) a very rare autosomal recessive disorder with an estimated prevalence of < 1 in 1000000^27–31^. The phenotypic spectrum encompasses neonatal lethal osteosclerotic bone dysplasia with respiratory distress and nonlethal forms of various severity. Typical features of the latter comprise hypophosphatemia, neurological disorders, midface hypoplasia, exophthalmos, depressed nasal bridge and various orodental anomalies including AI, gingival overgrowth and ectopic calcifications^32–35^. To date, 42 *FAM20C* disease-causative variants, 22 lethal and 20 nonlethal, have been described^29,36^. They include whole gene deletion, microdeletions, missense, nonsense and splice-site variants. Variants that affect protein stability and/or secretion or abolish the kinase activity are generally lethal whereas residual activity is compatible with life^15,37^.

We previously reported the systemic and orodental features of two consanguineous Brazilian families both presenting with AI, facial dysmorphism and hypophosphatemia^32^. Genetic analysis identified two distinct homozygous disease-causative variants in *FAM20C,* confirming nonlethal forms of RNS. The P496L substitution (Patient RNS-1) probably disrupting the FAM20C activation loop was associated with hypophosphatemic rickets, intracranial calcifications, visual impairment and an oral phenotype characterized by AI, dentinal anomalies and gingival hyperplasia with calcifications. The W202Cfs* splice site variant (Patient RNS-2) most likely produced a truncated protein of 239 amino acids and low levels of the wild-type protein presumably creating a hypomorphic allele. Mildly dysmorphic facies, intracranial calcifications, hearing impairment, AI, impaired dentin formation and gingival calcifications were observed^32^. Among the orodental features AI is the best studied in nonlethal RNS; moreover the specific role of FAM20C in amelogenesis has been established in murine models and *in vitro*^32,34,38–41^. On the contrary much less is known about the alterations of the RNS gingiva: gingivitis, gingival hypertrophy and gingival hyperplasia are the terms used in the literature to describe the gingival phenotype of the patients^32,34,35^.

Using conditioned media from primary gingival fibroblast (GF) cultures we previously characterized the secretome of ERS-derived GFs^42^. We identified TGFβ as a pathogenetic factor for ERS gingival fibrosis and showed that FAM20A and FAM20C were co-expressed in the human gingiva^8,42^. Given the described FAM20A/FAM20C interactions^3^ we wondered whether FAM20C dysfunction would also result in similar molecular and signaling modifications and/or gingival phenotype. To provide a precise description of the RNS-associated gingival phenotype and gain insight into the underlying pathogenetic mechanism(s) we here combined immunomorphological and biochemical analyses of RNS gingiva and RNS gingiva-derived GFs (RNS-GFs). We used a liquid chromatography tandem-mass-spectrometry-based label-free quantitative proteomic approach (LC–MS/MS) to analyze the proteome of control and RNS-GF lysates and furthered our investigation of the secretome of the same cell types. The protein signature of RNS-1 and RNS-2 cellular proteomes and secretomes were differentially compared to control samples. Variant-specific, albeit subtle differences were observed in the number of dysregulated proteins and cellular functions. ECM disorganization and impaired ossification were common findings in RNS-1 and RNS-2 whereas cytoskeletal modifications were mainly associated with RNS-2. Subsequent RT-PCR and biochemical analyses confirmed the proteomic data and revealed that TGFβ signaling was abnormally increased in the mutant gingival tissue and cells. Increased TGFβ signaling promoted YAP/TAZ signaling most likely creating a profibrotic loop consistent with persistent gingival fibrosis. The TGFβ-YAP/TAZ-FAM20C interplay was further confirmed in FAM20C- depleted human GFs. In sum, our data clearly define the gingival defects of RNS patients, provide the first to our knowledge proteomic analysis of RNS-GFs and uncover the previously unrecognized role of TGFβ–YAP/TAZ signaling in the pathogenesis of the gingival fibrosis in RNS.

## Results

### FAM20A and FAM20C expression in the control and RNS gingivas

**Figure 1:**
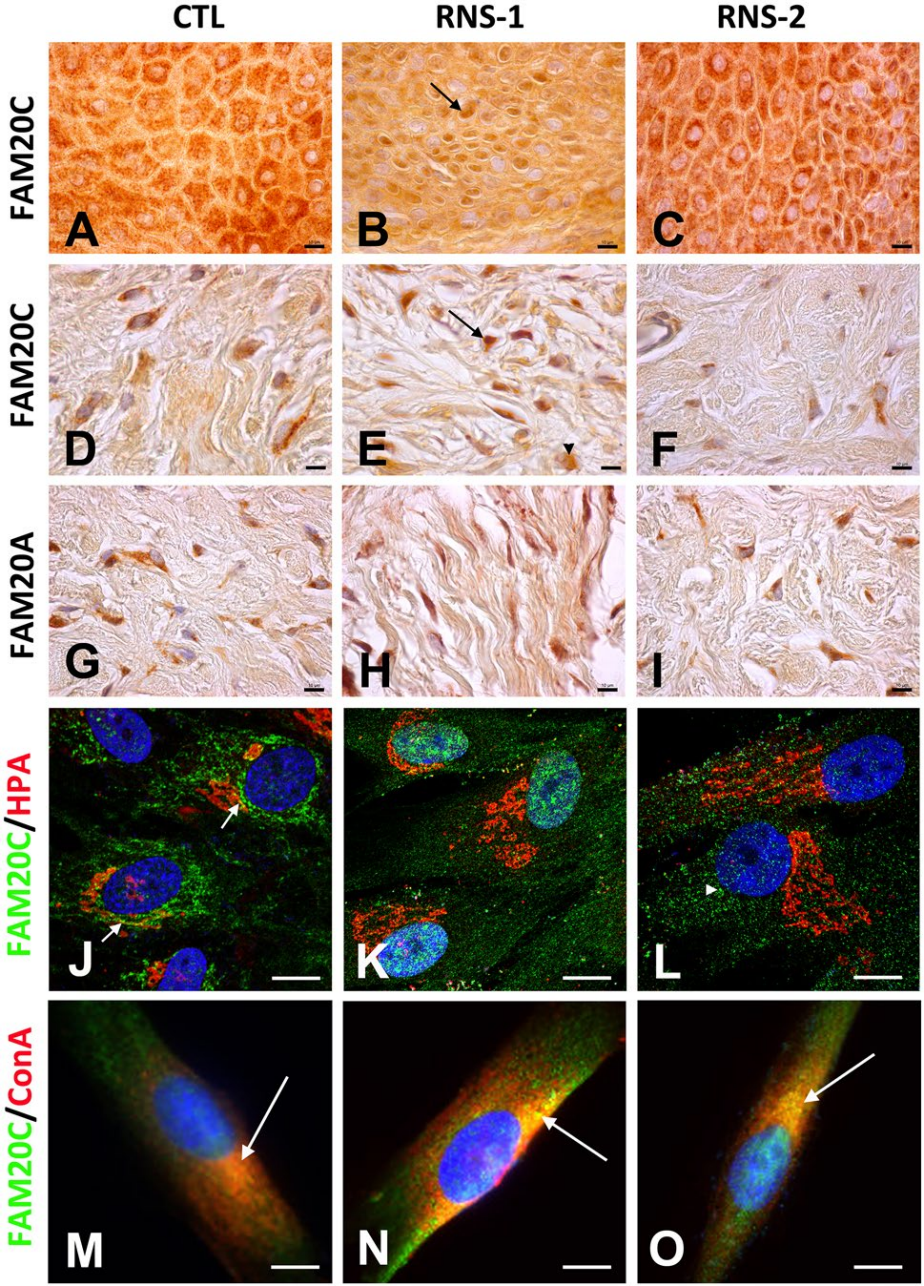
Distribution of FAM20C and FAM20A in control and RNS mutant gingivas. (**A**,**D**) In control gingiva, FAM20C immunoreactivity was found in large cytoplasmic vesicles of keratinocytes (**A**) and fibroblasts (**D**). (**B**,**E**) In RNS-1 gingiva, FAM20C immunoreactivity was localized around the nuclei of keratinocytes (arrow, (**B**)) and fibroblasts (arrow, (**E**)). Diffuse cytoplasmic immunoreactivity was also observed in some cells (arrowhead). (**C**,**F**) In RNS-2 gingiva, FAM20C was normally found in the cytoplasm of both keratinocytes (**C**) and fibroblasts (**F**), albeit with a weaker staining. (**G**–**I**) FAM20A was expressed in the cytoplasm of fibroblasts of control (**G**) as well as RNS-1 (**H**) and RNS-2 (**i**) gingivas. (**J**) In GFs FAM20C expression (green) was found in the endoplasmic reticulum and at the interface with the *cis*-Golgi in *HPA*-positive (red) compartment (arrows). (**K**) In RNS-1 GFs, the staining was perinuclear. (**L**) In RNS-2 GFs, the staining was weaker and appeared in small cytoplasmic aggregates (arrowhead). (**M**–**O**) Co-staining of control, RNS-1 and RNS-2 GFs with Concanavalin A. (**M**) Limited co-localization (arrow) of FAM20C (green) and the ER marker Concanavalin A in control GFs. (**N**) Extensive colocalization (arrow) of FAM20C and Concanavalin A in RNS-1 GFs. (**O**) Extensive colocalization (arrow) of FAM20C and Concanavalin A in RNS-2 GFs. Scale bars: (**A**–**I**) = 30 μm; (**J**–**O**) = 12 μm.

The co-expression of FAM20A and FAM20C in the human gingival keratinocytes, endothelial cells, macrophages and fibroblasts was previously reported^8^. In agreement with these data, FAM20C and FAM20A were readily detected and displayed a similar pattern in the control gingival keratinocytes and connective tissue (Fig. 1A,D,G and Supplementary Fig. 1A). The FAM20C signal was vesicular and perinuclear especially in the RNS-1 gingival cells (Fig. 1B,E,C,F). FAM20A distribution was not modified in the mutant tissues (Fig. 1G–I and Supplementary Fig. 1A). In vitro, FAM20C displayed a scattered vesicular pattern; in the mutant GFs a perinuclear distribution of the signal was also observed (Fig. 1J–L and Supplementary Fig. 1B). Whereas in control GFs FAM20C could be seen in HPA-positive cis-Golgi vesicles (Fig. 1J), no co-localization between FAM20C and HPA was evidenced in the RNS gingivas (Fig. 1K,L). We further analysed the subcellular distribution of FAM20C using the endoplasmic reticulum (ER) marker concanavalin A, the nuclear marker lamin-B1, the lysosomal marker Lamp1 and the ubiquitin–proteasome system associated protein UCHL1 known to be localized in the cytosol and the nucleus^43^ (Fig. 1 M–O and Supplementary Fig. 1C–K). Fam20C was readily identified in ER structures especially in the patients’ GFs. No co-localization could be seen with the nuclear or lysosomal markers either in control or patients’ GFs.

### Immunohistological analysis of RNS gingival tissue

**Figure 2:**
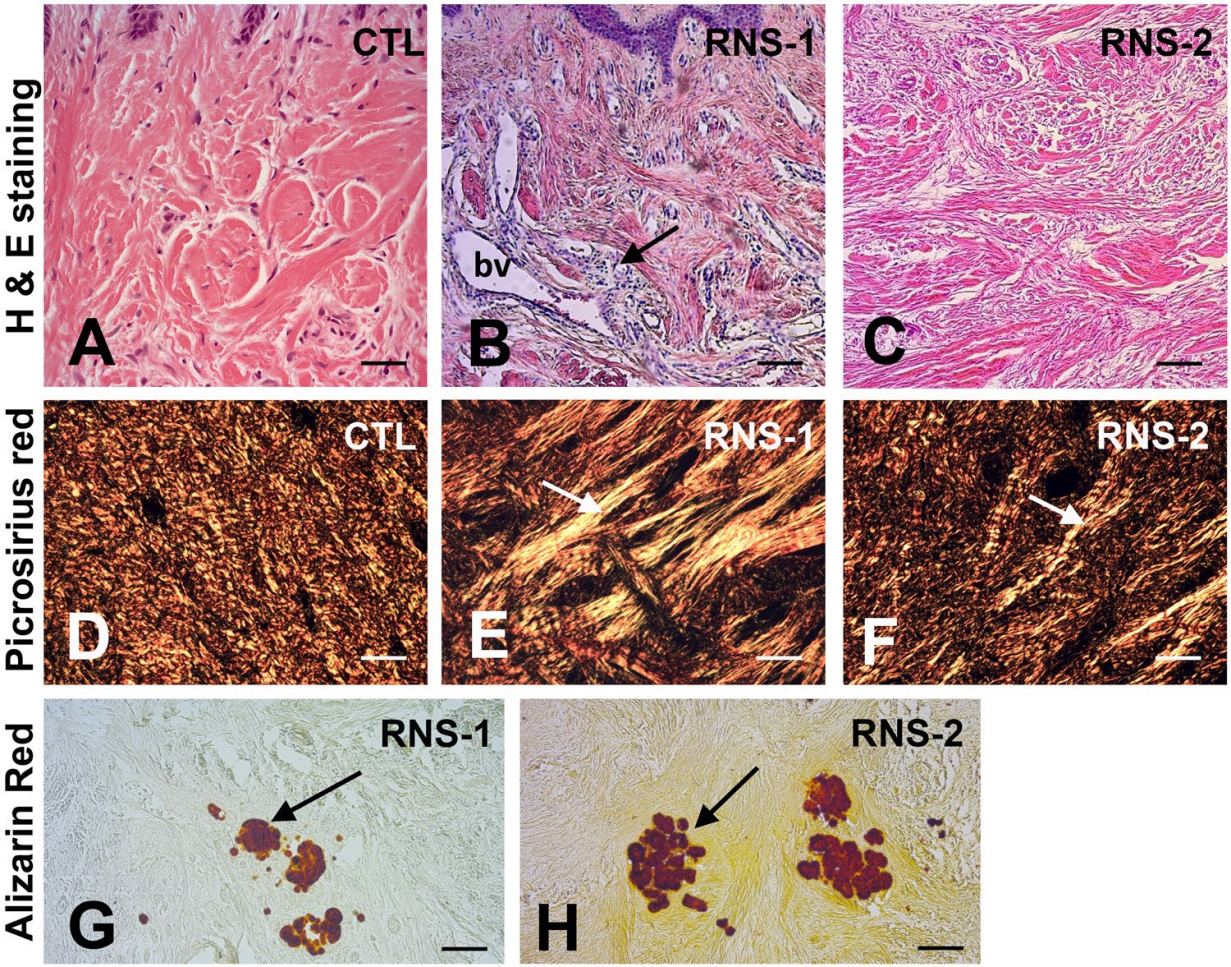
Histologic features of RNS variants. (**A**–**C**) Hematoxylin–eosin staining showed numerous tortuous blood vessels (bv) with an abnormally large diameter in the papillary layer and associated extensive inflammatory infiltrates in RNS-1 gingiva (**B**). Abnormally large and shredded collagen bundles running in all directions were found in RNS-2 gingiva (**C**). (**D**–**F**) Picrosirius red polarizing staining. (**D**) Fibers of collagen organized in bundles of fixed diameter running in perpendicular pathways. (**E**,**F**) In RNS-1 and RNS-2, collagen fibers did not organize in packed bundles, but appeared shredded (arrow) and organized in a mesh network. (**G**,**H**) Alizarin red staining identified calcium aggregates (arrow) in the reticular layer of the lamina propria of RNS-1 (**G**) and RNS-2 (**H**) mutants. Scale bars: (**A**–**C**), (**G**,**H**) = 200 μm; (**D**–**F**) = 50 μm.

The presence of gingival calcifications in the patients was previously reported^32^. Hematoxylin–eosin staining showed epithelial acanthosis and inflammatory infiltrates exclusively in RNS-1 and RNS-2 samples (Fig. 2A–C; Supplementary Fig. 2A–C). The vascular network was overdeveloped, particularly in RNS-1 (Fig. 2B and Supplementary Fig. 2B) and the connective tissue contained disorganized collagen fibre bundles (Fig. 2A–C). Picrosirius red polarizing staining confirmed this observation and showed that thick orange-red, presumably Collagen I, fibers were irregularly distributed in the mutant connective tissues (Fig. 2D–F). Collagen distribution was further evidenced by immunostaining (Supplementary Fig. 2D–F). Small, calcified aggregates were seen in the connective tissue in both RNS-1 and RNS-2 samples (Fig. 2G,H) but not the control gingivas.

### Cell proliferation, cell death and ectopic calcifications in RNS-GFs

Primary GF cultures were obtained from unrelated controls and the above described RNS patients. Western blot analysis of control and mutant cell extracts showed similar expression levels of PCNA and Annexin A1 in control and RNS- GFs indicating that cell proliferation and apoptosis were not affected (Supplementary Fig. 3).

**Figure 3:**
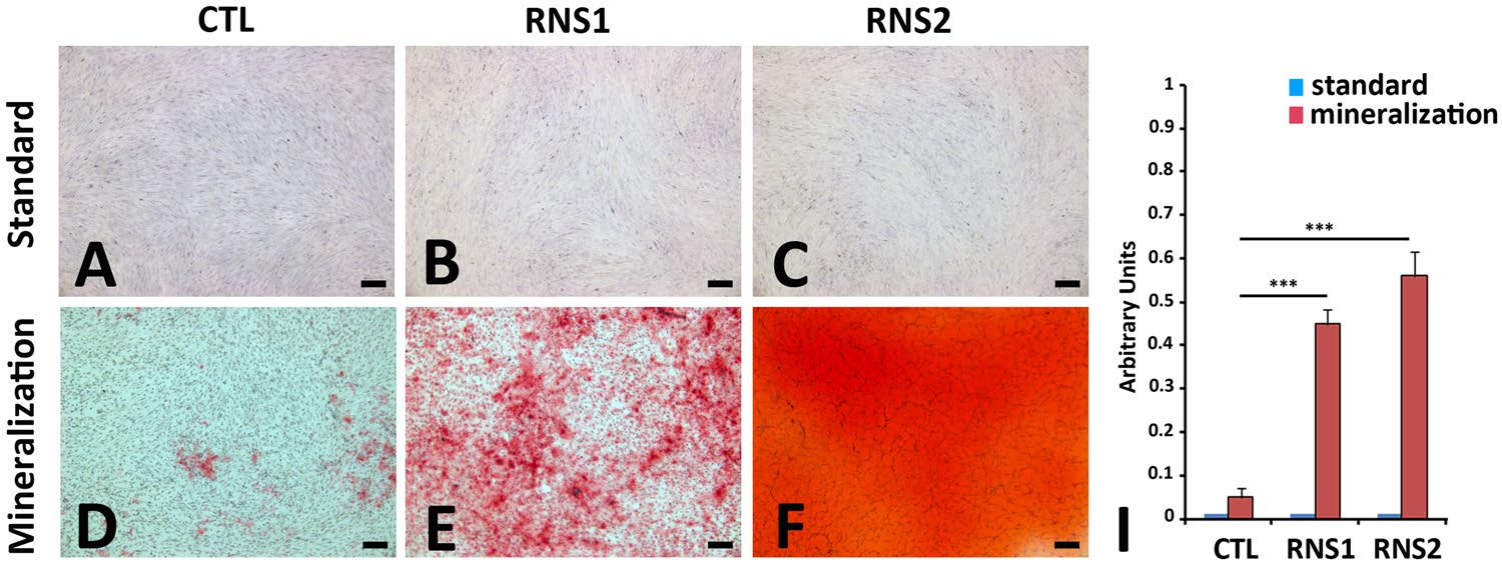
Mineralization in vitro. (**A**–**D**) Representative bright field images of normal (**A**), and FAM20C RNS (**B**,**C**) deficient GFs grown for 21 days in standard medium colored with Alizarin Red. (**D**–**F**) Representative bright field images of normal (**D**) and FAM20C (**E**,**F**) deficient GFs grown for 21 days in a mineralization-inducing medium colored with Red Alizarin. Arrowheads indicate rare and small calcium deposits in controls GFs. (**I**) Quantitative analysis of Alizarin Red expression by morphometric analysis on the pooled results of three separate experiments; the value represents the means ± SD. ***p < 0.001. Scale bars: (**A**–**F**) = 20 μm.

For mineralization assays, cells were cultured at passage 2 for a total period of 21 days in control and mineralization-inducing media as previously described ERS^8^. Figure 3 shows control, RNS-1- and RNS-2-derived GFs cultured in standard and mineralization-inducing media for 21 days. Alizarin red staining showed deposits of calcium aggregates in the mutant fibroblasts (Fig. 3E,F). In control GFs deposits were occasionally seen (Fig. 3D). Morphometric analysis showed a time dependent trend; calcium deposition significantly increased between 7 and 21 days of culture (Fig. 3I). This set of results suggested that FAM20C dysfunction may favor the osteogenic modifications of the mutant gingiva and ectopic calcification.

### Mass spectrometry analysis

**Figure 4:**
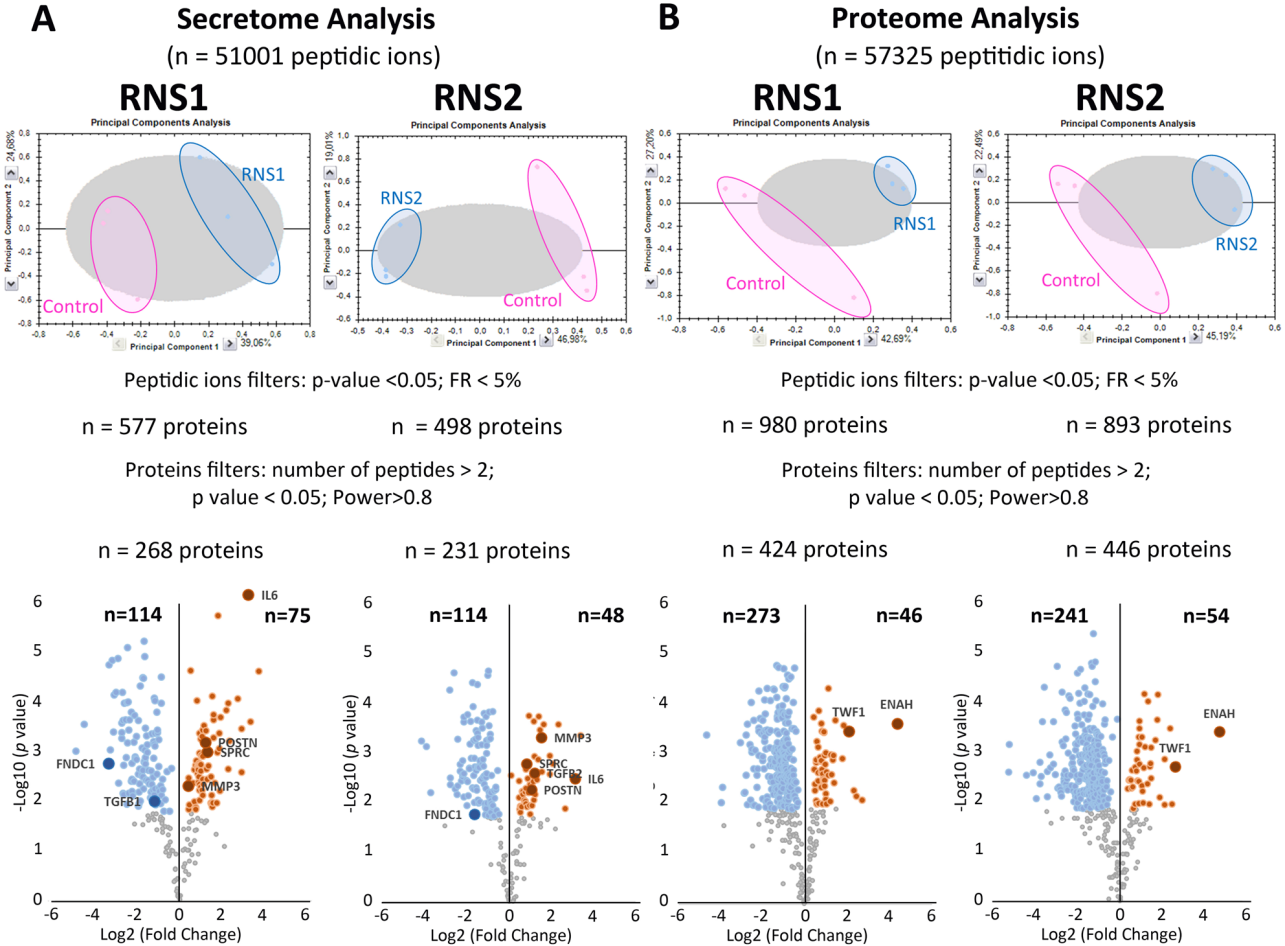
Mass spectrometry analysis. (**A**) Differential quantitative analysis between RNS1 (n = 3), RNS2 (n = 3) and Control (n = 3) on secretome. (B) Differential quantitative analysis between RNS1 (n = 3), RNS2 (n = 3) and Control (n = 3) on both cell proteome. By providing different statistical filters (Peptidic ions filters and proteins filters), the number of identified and quantified proteins are mentioned. A volcano plot, highlighting down- (blue circle) and up- (orange circle) regulated proteins, is presented for each comparison.

The GFs were cultured in standard conditions as described in the “Methods” section. Nano-LC–MS/MS was performed on conditioned media and cell lysates for secretome, and cellular proteome analysis respectively. To evaluate the reproducibility of the experiments, different linear regressions were performed by plotting the logarithm of protein intensities for the different samples of the same group (as mentioned in the “Methods” section). The averaged regression coefficient measured to evaluate the robustness of the technical scenario between biological replicates for the different groups of samples was estimated as follows: for cellular proteomes RCtls = 0.9954 and RRNS1/RNS2 = 0.9994; for secretome RRCtls = 08,835, RRNS1/RNS2 = 0.7938. The high regression coefficient of the samples analyzed allowed a reliable comparison of the control and mutant secretomes and cellular proteomes. For each comparison, a Principal Component Analysis on all peptidic ions showed a good discrimination of the 2 populations (Fig. 4A,B). By providing different statistical filters (peptide ions filters and proteins filters), the number of identified and quantified proteins was mentioned. A volcano plot, highlighting up- (orange circle) and down- (blue circle) regulated proteins, is presented for each comparison with a particular focus on proteins discussed and tested by RT-PCR (see further down).

In the secretome, 577 (RNS-1) and 498 (RNS-2) differentially expressed proteins were identified (Fig. 4A and Supplementary Table S1). Using criteria described in material and methods (at least two different peptides found; Anova p-value < 0.05; and power > 0.8), 268 (RNS-1) and 231 (RNS-2) proteins were significantly differentially expressed (Supplementary Table S2) compared with the controls. In RNS-1, 114 were underrepresented and 75 were overrepresented; in RNS-2, 114 were underrepresented and 48 were overrepresented (Supplementary Table S2). Taken together, 65 proteins were found in common between RNS-1 and RNS-2, among them 47 were underrepresented and 18 overrepresented (Supplementary Fig. 4, Supplementary Table S2). Further analysis showed that 55 of the 65 dysregulated proteins were ECM components (Supplementary Table S2).To identify how these up- and down-regulated proteins assembled into functional networks we used the Markov cluster algorithm http://www.micans.org/mcl/ with a 3.2 inflation parameter provided by the string-db.org (Supplementary Fig. 5 and Supplementary Table S3). We identified a major cluster of 44 COL1-associated secreted ECM proteins (Supplementary Fig. 5). This cluster was centered on the overrepresented COL1A1, COL1A2, COL5A2, IL-6, POSTN, SPARC, VIM, MMP3, SERPINE1, THSB1 and THSB2 (nodes in blue/grey circles) and the underrepresented FN1, COL12A1, BGN, COL15A1, LAMA4, LAMB2, LAMC1, TIMP1 and MMP1 (nodes in red/rose circles). ECM organization, regulation of cell-substrate adhesion and response to wound healing (Table 1) were the most enriched biological processes. GO analysis of the molecular functions showed a very strong enrichment in ECM structural constituents. Molecular functions related to the collagen-containing ECM were significantly enriched (Table 1). We used the Monarch Initiative, an integrative data and analytic platform connecting phenotypes to genotypes to integrate our data into functions (Table 2). Not surprisingly, in this group of proteins, most Monarch annotations corresponded to genes associated with skin wound healing or bone morphology and density (Table 2). GO disease analysis showed that among diseases, connective tissue disease was highly significant (Table 2). To identify specific functions that could be masked by a high number of genes, we also performed the same clustering analysis with individual RNS secretomes (Supplementary Tables S4, S5, S6 and S7). Cluster analysis of the dysregulated proteins in RNS-1 showed that 4 clusters of more than 6 proteins were formed (Supplementary Table S4). GO, Monarch analysis of the main cluster of 83 proteins indicated almost the same annotations as those mentioned above (Supplementary Fig. 6 and Supplementary Table S5). Cluster analysis of the proteins dysregulated in RNS-2 identified 4 clusters of more than 6 proteins (Supplementary Fig. 7 and Supplementary Table S6). As above, the main cluster of 63 proteins displayed similar annotations (Supplementary Table S7).

**Table 1:** Most enriched GO terms in RNS secretomes: biological process, molecular function, cellular component.

	Name	p value	Genes from input	Genes in annotation
Biological process				
GO:0030198	Extracellular matrix organization	1.85e–26	24	3338
GO:0010810	Regulation of cell-substrate adhesion	5.96e–09	11	212
GO:0051093	Negative regulation of developmental process	5.96e–09	18	983
GO:0051241	Negative regulation of multicellular organismal process	1.32e–08	19	1231
GO:0009611	Response to wounding	1.63e–08	14	532
GO:0042060	Wound healing	2.17e–08	13	439
GO:0030155	Regulation of cell adhesion	4.04e–08	15	712
GO:0032963	Collagen metabolic process	2.41e–07	7	63
GO:0050793	Regulation of developmental process	2.41e–07	24	2448
GO:0030334	Regulation of cell migration	4.25e–07	15	865
Molecular function				
GO:0005201	Extracellular matrix structural constituent	6.76e–17	14	119
GO:0005198	Structural molecule activity	2.90e–11	17	635
GO:0005518	Collagen binding	3.32e–07	7	68
GO:0061134	Peptidase regulator activity	1.74e–06	9	226
GO:0030020	Extracellular matrix structural constituent conferring tensile strength	6.59e–06	5	28
GO:0005539	Glycosaminoglycan binding	3.77e–05	8	240
GO:0050840	Extracellular matrix binding	0.00011	5	56
GO:0002020	Protease binding	0.00032	6	138
GO:0004866	Endopeptidase inhibitor activity	0.00098	6	181
GO:0004867	Serine-type endopeptidase inhibitor activity	0.00098	5	98
Cellular component				
GO:0031012	Extracellular matrix	3.29e–35	31	527
GO:0062023	Collagen-containing extracellular matrix	1.88e–31	27	396
GO:0005576	Extracellular region	2.42e–23	42	4166
GO:0005615	Extracellular space	1.57e–21	38	3195
GO:1903561	Extracellular vesicle	3.62e–13	27	2121
GO:0005788	Endoplasmic reticulum lumen	1.40e–12	14	308
GO:0005604	Basement membrane	6.24e–12	10	96
GO:0070062	Extracellular exosome	2.19e–11	25	2099
GO:0031982	Vesicle	9.84e–11	31	3879
GO:0005581	Collagen trimer	9.72e–06	6	88

Selection based on p-value. Hit count in genome shows the number of genes in a given pathway, and the hit count in query list shows how many genes in the query list are hit in a given GO terms. The full output table generated by String.dg.org is shown in Supplementary Tables S3 and S4.

**Table 2:** Most enriched MONARCH and disease terms in RNS secretomes.

	Name	pValue	Genes from input	Genes in annotation
Monarch				
HP:0001075	Atrophic scars	4.44e–10	8	35
HP:0000977	Soft skin	1.97e–08	7	35
HP:0002808	Kyphosis	1.97e–08	13	406
HP:0100699	Scarring	1.97e–08	10	162
HP:0001058	Poor wound healing	3.95e–08	6	20
HP:0000592	Blue sclerae	4.01e–08	8	83
HP:0004348	Abnormality of bone mineral density	4.01e–08	13	469
HP:0008780	Congenital bilateral hip dislocation	7.08e–08	5	8
HP:0002645	Wormian bones	7.62e–08	7	54
HP:0002644	Abnormality of pelvic girdle bone morphology	1.21e–07	12	419
HP:0002814	Abnormality of the lower limb	1.27e–07	19	1509
HP:0000974	Hyperextensible skin	1.33e–07	7	63
HP:0011025	Abnormal cardiovascular system physiology	1.63e–07	17	1168
HP:0001030	Fragile skin	2.01e–07	6	34
HP:0001374	Congenital hip dislocation	2.60e–07	7	72
Disease				
DOID:65	Connective tissue disease	1.58e–12	19	715
DOID:17	Musculoskeletal system disease	7.68e–11	20	1074
DOID:13359	Ehlers-Danlos syndrome	5.95e–08	6	23
DOID:0080001	Bone disease	1.17e–06	12	523
DOID:7	Disease of anatomical entity	1.17e–06	29	4452
DOID:12347	Osteogenesis imperfecta	8.66e–06	5	30

Selection based on p-value. Hit count in genome shows the number of genes in a given pathway, and the hit count in query list shows how many genes in the query list are hit in a given terms. The full output table generated by String.dg.org is shown in Supplementary Tables S3 and S4.

**Figure 5:**
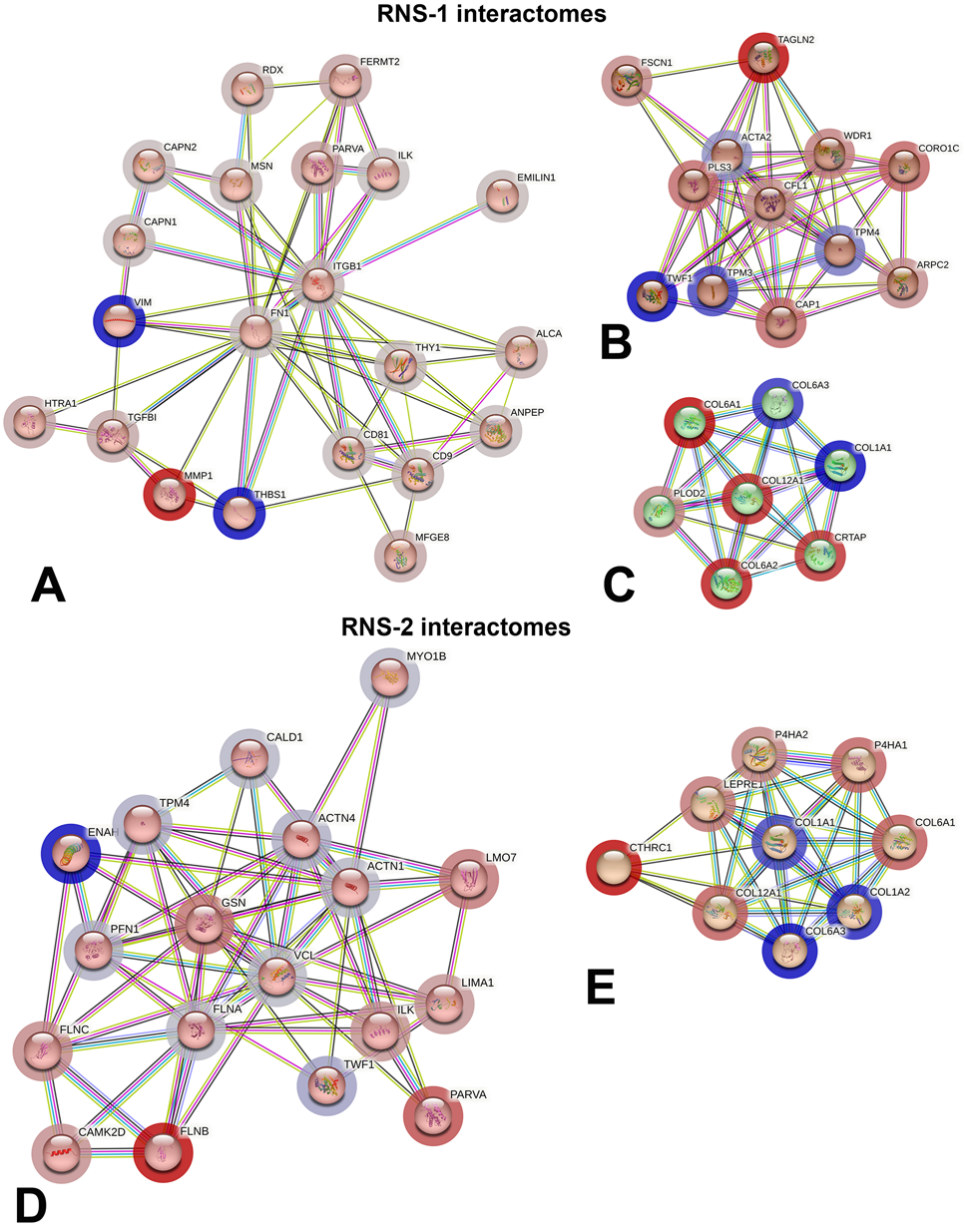
Protein–protein association network using String analysis performed with the differentially selected clustered proteins, over-expressed and under-expressed in the RNS proteomes. (**A**–**C**) Main interactomes of RNS-1 compared to controls, concerning extracellular matrix organization (**A**), actin filament organization (**B**) and collagen fibril organization (**C**). (**D**,**E**) Main interactomes of RNS-2 compared to controls, concerning actin skeleton organization (**D**) and collagen fibril organization (**E**). Nodes in blue/grey circles stand for overrepresented proteins; Nodes in red/grey circles stand for underrepresented proteins. Code color clusterisation, protein names, identifications and descriptions are provided in Tables S11 and S13.

In the cellular proteome 980 (RNS-1) and 893 (RNS-2) differentially expressed proteins were identified compared with the controls (Fig. 4B and Supplementary Table S8); of these proteins 424 (in RNS-1) and 446 (in RNS-2) were significantly modified (Supplementary Table S9). 188 dysregulated proteins were found in common in RNS-1 and RNS-2. Out of these proteins 25 were more abundant and 163 less abundant (Supplementary Fig. 4 and Supplementary Table S10) compared with the controls. All over- and underrepresented proteins were further analyzed using the Markov Cluster algorithm http://www.micans.org/mcl/ with a 3.2 inflation parameter provided by string-db.org. This analysis failed to give any relevant association; the statistical power of GO annotated processes, molecular functions or compartments was indeed low (Supplementary Table S10). Individual, variant-specific analysis was performed using the same algorithm. Cluster analysis of the dysregulated proteins in RNS-1 indicated 15 clusters of more than 6 proteins (Supplementary Table S11). These clusters essentially concerned mRNA translation, mRNA splicing, carboxylic acid metabolic process (Table S12). We focused on three clusters associated with cell-to-cell, cell-ECM interactions, collagen and actin cytoskeleton organization (Tables 3, 4; Supplementary Table S12). The first one was centered on the overrepresented VIM and THBS1 and the underrepresented FN1 and MMP1 (nodes in blue/grey circles in Fig. 5A–C). The second one comprised the overrepresented ACTA, TWF1, TPM3 and the underrepresented FSCN1 and TAGLN2 (nodes in red/rose circles in Fig. 5A–C). The third one was centered on the overrepresented COL1A1 and COL6A3 and the underrepresented COL12A1, COL6A1 and COL6A2.

**Table 3:** Most enriched GO terms in RNS-1 cellular proteome concerning extracellular matrix cluster: biological process, molecular function, cellular component.

	Name	pValue	Genes from input	Genes in annotation
Biological process				
GO:0009653	Anatomical structure morphogenesis	8.11e–11	18	2165
GO:0022610	Biological adhesion	1.84e–10	14	931
GO:0048646	Anatomical structure formation involved in morphogenesis	2.19e–09	13	883
GO:0007155	Cell adhesion	2.94e–09	13	925
GO:0030198	Extracellular matrix organization	9.71e–08	9	338
GO:0048856	Anatomical structure development	1.93e–07	20	5402
GO:0031589	Cell-substrate adhesion	1.03e–06	7	182
GO:0001525	Angiogenesis	1.09e–06	8	315
GO:0030154	Cell differentiation	1.67e–06	17	3702
GO:0016043	Cellular component organization	3.23e–06	19	5447
Molecular function				
GO:0005178	Integrin binding	7.04e–15	11	147
GO:0050839	Cell adhesion molecule binding	2.66e–14	14	538
GO:0044877	Protein-containing complex binding	3.01e–08	13	1216
GO:0005102	Signaling receptor binding	5.80e–07	13	1581
GO:0005518	Collagen binding	0.00062	4	68
GO:0005515	Protein binding	0.0021	18	7026
GO:0005201	Extracellular matrix structural constituent	0.0038	4	119
GO:0050840	Extracellular matrix binding	0.0134	3	56
GO:0005198	Structural molecule activity	0.0141	6	635
GO:0098634	Cell–matrix adhesion mediator activity	0.0141	2	7

	Name	pValue	Genes from input	Genes in annotation
GO:0005925	Focal adhesion	4.69e–14	13	405
GO:0005576	Extracellular region	9.58e–08	18	4166
GO:0009897	External side of plasma membrane	1.92e–07	8	331
GO:0098552	Side of membrane	2.67e–07	9	531
GO:0009986	Cell surface	5.19e–07	10	824
GO:0031252	Cell leading edge	2.29e–05	7	425
GO:0031012	Extracellular matrix	8.97e–05	7	527
GO:0030027	Lamellipodium	0.00021	5	202
GO:0062023	Collagen-containing extracellular matrix	0.00026	6	396
GO:0034668	Integrin alpha4-beta1 complex	0.00088	2	3

Selection based on p-value. Hit count in genome shows the number of genes in a given pathway, and the hit count in query list shows how many genes in the query list are hit in a given GO terms. The full output table generated by String.dg.org is shown in Supplementary Tables S11 and S12.

**Table 4:** Most enriched GO terms in RNS-1 cellular proteome concerning the actin cytoskeleton cluster: biological process, molecular function, cellular component.

	Name	p value	Genes from input	Genes in annotation
Biological process				
GO:0007015	Actin filament organization	1.39e–13	10	254
GO:0008154	Actin polymerization or depolymerization	4.50e–07	5	57
GO:1902743	Regulation of lamellipodium organization	3.65e–05	4	47
GO:0030042	Actin filament depolymerization	0.00010	3	11
GO:0006928	Movement of cell or subcellular component	0.00065	8	1501
GO:0010591	Regulation of lamellipodium assembly	0.0016	3	35
GO:0032956	Regulation of actin cytoskeleton organization	0.0016	5	348
GO:1902745	Positive regulation of lamellipodium organization	0.0016	3	33
GO:0120032	Regulation of plasma membrane bounded cell projection assembly	0.0029	4	181
GO:0008064	Regulation of actin polymerization or depolymerization	0.0030	4	189
Molecular function				
GO:0003779	Actin binding	7.31e–12	10	438
GO:0051015	Actin filament binding	1.05e–10	8	199
GO:0005515	Protein binding	0.0030	12	7026
Component				
GO:0015629	Actin cytoskeleton	4.12e–14	11	477
GO:0070161	Anchoring junction	2.39e–06	8	820
GO:0005925	Focal adhesion	2.91e–05	6	405
GO:0030027	Lamellipodium	2.91e–05	5	202
GO:0032432	Actin filament bundle	2.91e–05	4	73
GO:0043292	Contractile fiber	4.43e–05	5	238
GO:0005884	Actin filament	5.87e–05	4	101
GO:0070062	Extracellular exosome	5.87e–05	9	2099
GO:0005938	Cell cortex	7.37e–05	5	292
GO:0031982	Vesicle	0.00042	10	3879

Selection based on p-value. Hit count in genome shows the number of genes in a given pathway, and the hit count in query list shows how many genes in the query list are hit in a given GO terms. The full output table generated by String.dg.org is shown in Supplementary Tables S11 and S12.

Cluster analysis of the dysregulated proteins in RNS-2 identified 15 clusters of more than 6 proteins (Supplementary Table S13). As in RNS-1, clusters of proteins involved in mRNA splicing and carboxylic acid metabolic processes were seen (Supplementary Table S14). We focused on two clusters; the first one was involved in actin filament organization and comprised the overrepresented ENAH, TWF1, TPM4, ACTN4 and ACTN1 (nodes in blue/gray circles in Fig. 5D,E) and the underrepresented GELS, FLNB and PARVA (nodes in red/rose circles in Fig. 5D,E). The second one was centered on the overrepresented COL1A1, COL1A2, COL6A3 and the underrepresented COL6A1 and COL12A1 (Fig. 5E, Tables 5, 6, and Supplementary Table S14).

**Table 5:** Most enriched GO terms in RNS-2 cellular proteome concerning actin cytoskeleton cluster: biological process, molecular function, cellular component.

	Name	pValue	Genes from Input	Genes in Annotation
Biological process				
GO:0030029	Actin filament-based process	1.96e–12	13	592
GO:0030036	Actin cytoskeleton organization	1.34e–11	12	516
GO:0007015	Actin filament organization	2.32e–09	9	254
GO:0097435	Supramolecular fiber organization	1.03e–08	10	480
GO:0032970	Regulation of actin filament-based process	7.50e–05	7	390
GO:0030048	Actin filament-based movement	0.00015	5	121
GO:0034329	Cell junction assembly	0.00024	6	280
GO:0034330	Cell junction organization	0.00024	7	493
GO:0051017	Actin filament bundle assembly	0.00028	4	55
GO:0000904	Cell morphogenesis involved in differentiation	0.00046	7	566
Molecular function				
GO:0003779	Actin binding	5.72e–19	15	438
GO:0008092	Cytoskeletal protein binding	3.59e–16	16	973
GO:0050839	Cell adhesion molecule binding	3.67e–12	12	538
GO:0045296	Cadherin binding	4.40e–09	9	334
GO:0051015	Actin filament binding	4.40e–09	8	199
GO:0005515	Protein binding	0.00018	17	7026
GO:0044877	Protein-containing complex binding	0.00019	9	1216
GO:0005546	phosphatidylinositol-4,5-bisphosphate binding	0.00034	4	78
GO:0003785	Actin monomer binding	0.0010	3	28

	Name	pvalue	Genes from input	Genes in annotation
GO:0005925	Focal adhesion	9.53e–20	15	405
GO:0015629	Actin cytoskeleton	4.18e–15	13	477
GO:0030016	Myofibril	6.80e–15	11	227
GO:0005856	Cytoskeleton	2.37e–11	16	2221
GO:0099512	Supramolecular fiber	4.30e–10	12	939
GO:0030017	Sarcomere	1.14e–09	8	207
GO:0001725	Stress fiber	4.65e–09	6	65
GO:0005903	Brush border	5.36e–08	6	106
GO:0030018	Z disc	1.72e–07	6	131
GO:0005938	Cell cortex	4.02e–07	7	292

Selection based on p-value. Hit count in genome shows the number of genes in a given pathway, and the hit count in query list shows how many genes in the query list are hit in a given GO terms. The full output table generated by String.dg.org is shown in Supplementary Tables S13 and S14.

**Table 6:** Most enriched GO terms in RNS-2 cellular proteome concerning ECM cluster: biological process, molecular function, cellular component.

	Name	pvalue	Genes from input	Genes in annotation
Biological process				
GO:0030198	Extracellular matrix organization	2.91e–05	6	338
GO:0030199	Collagen fibril organization	2.91e–05	4	46
GO:0060351	Cartilage development involved in endochondral bone morphogenesis	2.91e–05	4	43
GO:0060348	Bone development	3.66e–05	5	199
GO:0001501	Skeletal system development	4.82e–05	6	499
GO:0003429	Growth plate cartilage chondrocyte morphogenesis	0.00011	3	17
GO:0009887	Animal organ morphogenesis	0.00061	6	967
GO:0032963	Collagen metabolic process	0.0014	3	63
GO:0071230	Cellular response to amino acid stimulus	0.0017	3	69
GO:0001503	Ossification	0.0018	4	265
Molecular function				
GO:0030020	Extracellular matrix structural constituent conferring tensile strength	4.02e–09	5	28
GO:0019798	Procollagen-proline dioxygenase activity	8.69e–06	3	7
GO:0048407	Platelet-derived growth factor binding	1.97e–05	3	11
GO:0031418	L-ascorbic acid binding	6.39e–05	3	20
GO:0004656	Procollagen-proline 4-dioxygenase activity	0.0011	2	4
GO:0005506	Iron ion binding	0.0077	3	147

	Name	pvalue	Genes from input	Genes in annotation
GO:0005788	Endoplasmic reticulum lumen	6.37e–11	8	308
GO:0005581	Collagen trimer	7.36e–10	6	88
GO:0062023	Collagen-containing extracellular matrix	3.26e–06	6	396
GO:0005584	Collagen type I trimer	0.00032	2	2
GO:0005589	Collagen type VI trimer	0.00046	2	3

Selection based on p-value. Hit count in genome shows the number of genes in a given pathway, and the hit count in query list shows how many genes in the query list are hit in a given GO terms. The full output table generated by String.dg.org is shown in Supplementary Tables S13 and S14.

We used cell lysates of GFs cultured under standard conditions to analyze the transcript levels of several strongly dysregulated proteins including POSTN, SPARC, TWF1, MMP3, ENAH, FNDC1, a protein which contains a conserved protein domain of FN1 and IL6. Several of these proteins are known TGFβ targets and have previously been associated with fibrotic modifications^44^. We therefore also analyzed the levels of the TGFβ signaling actors TGFB1, TGFB2 and TGFBR2 as well as FAM20A and FAM20C.

**Figure 6:**
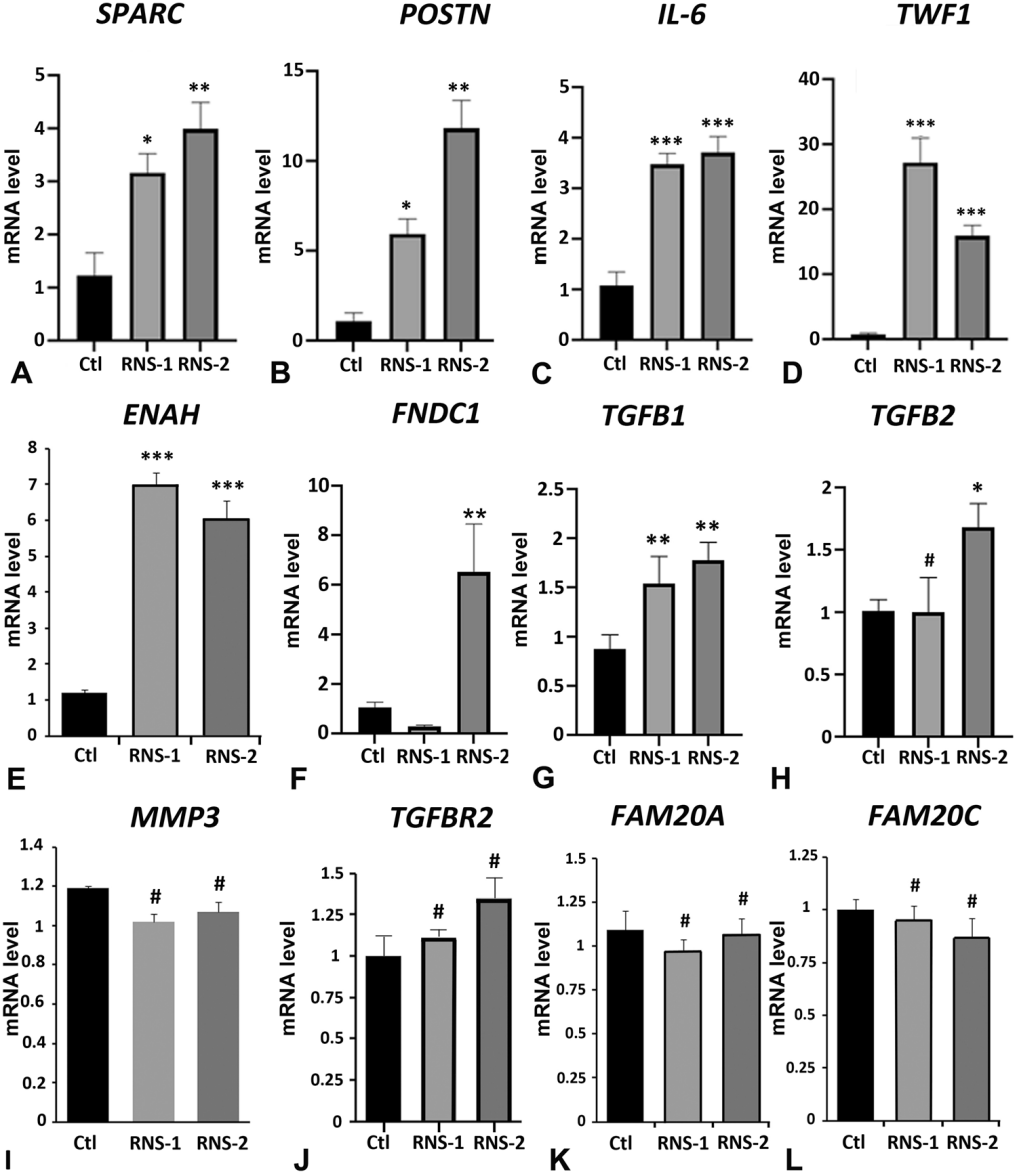
Real time RT-PCR analysis of candidate genes corresponding to proteins with differential abundance characterized in proteomic analysis and to proteins of the TGFβ pathway: *Osteonectin* ((**A**), *SPARC*), *Periostin* ((**B**), *POSTN*), *Interleukin-6* ((**C**), *IL6*), *Twinfillin-1* ((**D**), *TWF1*), *Protein Enabled Homolog* ((**E**), *ENAH*), *Fibronectin type III domain-containing protein 1* ((**F**), *FNDC1*), *TGFB1* (**G**), *TGFB2* (**H**), MMP3 (**I**), *TGFBR2* receptor (**J**), *FAM20A* (**K**) and *FAM20C* (**L**). Control values correspond to the mean of 3 independent experiments in triplicates of three control cultures. RNS values correspond to the mean of 3 independent experiments in triplicates of RNS patient cultures. Data represent mean fold gene expressions ± s.d. Data were analyzed via one-way ANOVA with Bonferroni multiple comparisons test (*p < 0.05, **p < 0.01, ***p < 0.001: ^#^not significant).

Consistent with the data of dysregulated proteins in secretomes and cellular proteomes, the mRNA levels for *SPARC*, *POSTN*, *IL6*, *TWF1*and *ENAH* were significantly albeit differentially increased in RNS-1 and RNS-2 samples (Fig. 6A–E). *FNDC1* synthesis was almost sixfold upregulated in RNS-2 but not significantly modified in RNS-1 despite very low protein levels in both secretomes (Fig. 6F). The *TGFB1* transcript was strongly upregulated in RNS-1- and RNS-2-GFs (Fig. 6G) whereas *TGFB2* was significantly increased in RNS-2-GFs (Fig. 6H). No significant differences were evident for *MMP3*, *TGFBR2*, *FAM20C* and *FAM20A* mRNA levels (Fig. 6I–L).

Taken together, the above data suggested that biological processes involved in ECM, actin cytoskeleton and stress fiber organization, response to wound healing and connective tissue disease were modified in the mutant GFs. These data were consistent with the fibrotic phenotype of the RNS gingiva shown in Fig. 2 and published data involving FAM20C in connective tissue disease^15,45,46^. They also strongly suggested that modifications in the TGFβ pathway may result from FAM20C dysfunction in the gingival tissue. We therefore subsequently investigated the expression of selected dysregulated proteins in vivo and analyzed their involvement in fibrotic processes.

### In vivo expression of selected dysregulated proteins

The secretome data showed that the expression of several TGFβ signaling targets including the profibrotic proteins POSTN, COL5A, VIM, SPARC and FN1 was significantly modified in RNS-GFs. To confirm the in vivo relevance of these data, we investigated the expression of the above proteins as well as of α-SMA, a key marker of fibroblast-to-myofibroblast differentiation and known TGFβ target^42,44,47–50^ in gingival tissues.

**Figure 7:**
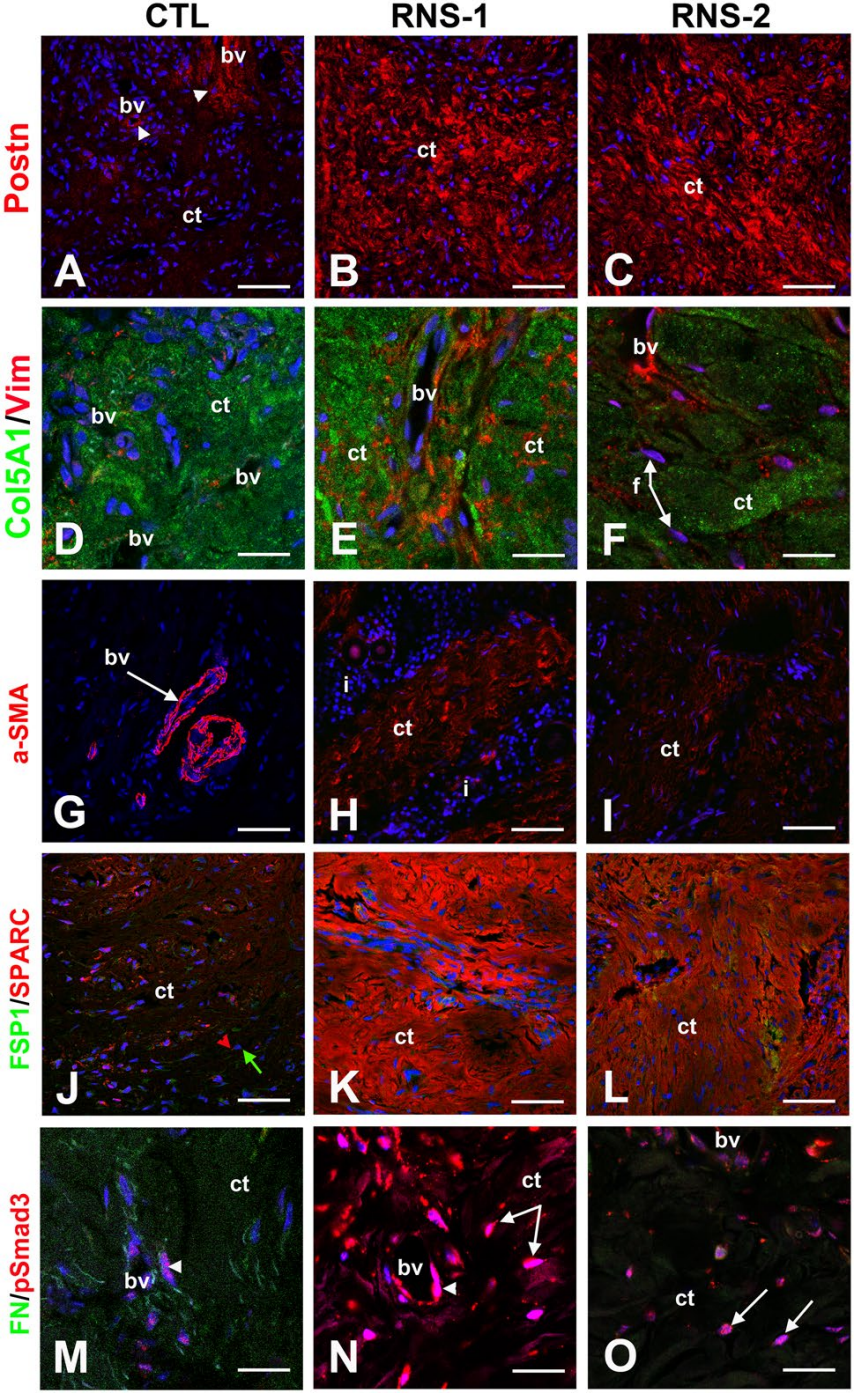
Immunocytochemical features of gingival connective tissues in the gingiva of RNS variants. (**A**) In normal gingiva, POSTN is localized in a loose extracellular network around blood vessels (arrowheads; bv) whereas in both RNS-1 (**B**) and RNS-2 (**C**) mutants POSTN extracellular expression is localized along thick and disorganized fibers in the connective tissue (ct). (**D**) In normal gingiva, Collagen 5 alpha-1 (green; Col5A1) is secreted and deposited along collagen 1 fibers and Vimentin (red; Vim) is expressed by endothelial cells. (**E**,**F**) Increased expression of Col5A1 is detected in the connective tissue (ct) of RNS-1 (**E**) and RNS-2 (**F**) mutants.Vim is normally localized in endothelial cells and abnormally in fibroblasts (**F**). (**G**) Alpha-SMA (α-SMA) is a specific marker of contractile endothelial cells of bv. (**H**,**I**) In RNS mutants, α-SMA is also expressed by fibroblasts in ct. Note the presence of inflammatory infiltrates (i) in RNS-1 gingiva. (**J**) In normal gingiva, Fibroblast-specific protein 1 (green; FSP1) is expressed by gingival fibroblasts (green arrow). Osteonectin (red; SPARC) is expressed in discrete spots by fibroblasts (red arrowhead) and endothelial cells. (**K**,**L**) FSP-1 is normally expressed in ct. SPARC expression and SPARC deposition along fibers are dramatically increased in RNS mutant connective tissues. (**M**) Fibronectin (green; FN) is specifically expressed by fibroblasts. Phospho-SMAD3 (red; pSMAD3) is mainly localized in nuclei of vascular cells (arrowhead). (**N**,**O**) In RNS-derived gingiva FN expression is dramatically decreased. pSMAD3 expression is dramatically increased in both vascular cells (arrowhead) and fibroblasts (arrows) nuclei. Scale bars: (**A**–**C**), (**G**–**L**) = 100 μm; (**D**–**F**), (**M**–**O**) = 30 μm.

The expression of POSTN, COL5A, VIM, α-SMA, SPARC and FN1 in RNS gingivas is shown in Fig. 7 (Fig. 7 and Supplementary Fig. 6). POSTN, a matricellular protein upregulated in remodeling tissues^51^ displayed a vesicular pattern in the control fibroblasts; in the RNS gingivas POSTN accumulated in the surrounding ECM (Fig. 7A–C). COL5A was similarly distributed in control and mutant tissues but VIM a key factor in fibrotic processes, was strongly expressed in the fibroblasts and/or myofibroblasts throughout the RNS gingivas (Fig. 7D–F).

Alpha-SMA, a hallmark of mature myofibroblasts is also normally expressed by vascular smooth muscle cells and/or pericytes^52^. A perivascular staining was observed for α-SMA in control and mutant gingivas (Fig. 7G-I). In addition, a particularly intense signal was seen in the mutant connective tissue, most likely associated with myofibroblasts (Fig. 7H,I). SPARC modulates cell–matrix interactions and is generally found in tissues undergoing remodeling^53^. SPARC was expressed by gingival fibroblasts, also positive for the fibroblastic marker FSP1 (Fig. 7J–L). In the RNS gingivas the staining was particularly strong and SPARC was seen both in the cytoplasm and the surrounding ECM (Fig. 7K,L).

A typical mediator of TGFβ signaling is Smad3; the phosphorylation and subsequent nuclear localization of pSmad3 indicate activation of the pathway^54^. In control and RNS gingivas pSmad3 was detected in vascular and fibroblastic cells (Fig. 7M–O). The nuclear localization of pSmad3 specifically in the RNS fibroblasts indicated hyperactivation of the TGFβ pathway in this cell type (Fig. 7M–O). In agreement with the proteomic data, FN1 was not detectable in the RNS gingivas (Fig. 7M–O).

### FAM20C dysfunction or knockdown increase TGFβ and YAP/TAZ signaling

**Figure 8:**
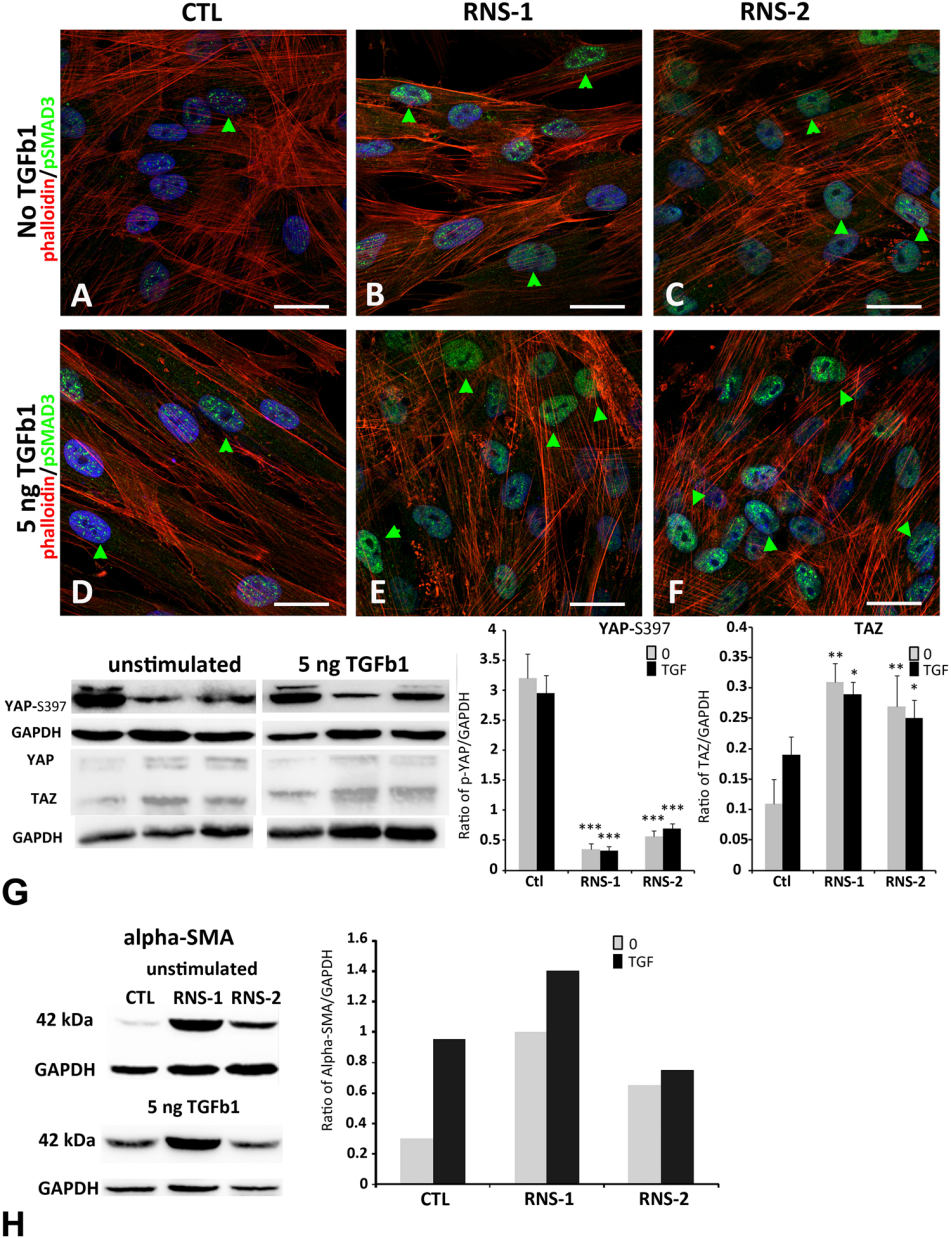
SMAD3 activation in untreated RNS GFs and treated control and RNS GFs. Immunocytochemical staining of control (**A**,**D**) and RNS (**B**,**C**,**E**,**F**) GFs cultured without TGFβ1 (**A**–**C**) or with 5 ng/ml TGFβ1 (**D**–**F**) for 6 h. Cells were fluorescently labeled for p-SMAD3 (green), nuclei (blue), and the specific F-actin marker, phalloidin (red). Co-localization of p-SMAD3 and nuclei indicated nuclear translocation of p-SMAD3 (green arrow). (**A**) In control untreated GFs, p-SMAD3 immunoreactivity is detected at low levels in some nuclei. (**B**,**C**) In RNS untreated GFs, p-SMAD3 was increased in intensity in all nuclei. (**D**–**F**) TGFβ1 induced an increase in p-SMAD3 nuclei in normal (**D**) and mutant GFs (**E**,**F**). (**G**) Western blot were performed on cell lysates. P-YAP (Ser 397) protein levels were decreased in RNS GFs without or with TGFβ1 compared to Control. TAZ protein levels were increased in RNS GFs compared to normal GFs. Densitometric analysis of Phospho-YAP and TAZ bands normalized to corresponding GAPDH bands. Data represent mean fold change in band intensity ± s.d. relative to GAPDH of 3 independent experiments in triplicates. Data were analyzed by one-way ANOVA with Bonferroni multiple comparisons test (**p < 0.01, ***p < 0.001). (**H**) Western blots were performed on cell lysates. Alpha-SMA protein levels were increased in controls GFs cultured with TGFβ1. Alpha-SMA protein levels were increased in RNS GFs cultured without or with TGFβ1 compared to control. Densitometric analysis of a-SMA bands normalized to corresponding GAPDH levels. Scale bars: (**A**-**F**) = 20 µm.

To further investigate the above data and highlight the potential role of the TGFβ pathway in the RNS-associated gingival phenotype we treated control and RNS GFs with TGFβ 1 (5 ng/ml for 6 h). pSmad3 was readily detected in the nuclei of untreated RNS-1- and RNS-2-GFs and only occasionally in control GFs (Fig. 8A–C and Supplementary Fig. 7). The addition of TGFβ 1 for 6 h resulted in nuclear localization of pSmad3 in the controls and further enhanced nuclear pSmad3 accumulation in the mutants (Fig. 8D–F and Supplementary Fig. 7).

It was shown that fibroblast responsiveness to TGFβ was favored by the Hippo pathway targets, the transcription cofactors YAP/TAZ^55–58^. YAP/TAZ activity is regulated by phosphorylation and subcellular localization. YAP phosphorylation on Ser397 promotes protein degradation^59^ whereas the dephosphorylated/active form translocates to the nucleus where it allows transcription of target genes.

Under control conditions the levels of YAP/TAZ were higher in mutant GFs (Fig. 8G). The addition of TGFβ 1 for 6 h increased YAP/TAZ levels in both control and mutant cells (Fig. 8G) suggesting activation of YAP signaling. This was also confirmed by the amount of pYAP (S397) which was dramatically decreased in both untreated and TGFβ 1-induced RNS-GFs (Fig. 8G).Activation of YAP/TAZ and TGFβ signaling favor myofibroblast differentiation and fibrotic remodeling^60^. The myofibroblast marker α-SMA was indeed strongly upregulated in untreated RNS-GFs and this was confirmed at the protein and mRNA levels (Fig. 8H). Treatment with TGFβ 1 for 6 h increased α-SMA protein expression in control and slightly in RNS-GFs (Fig. 8H). This set of data indicates that activation of YAP/TAZ and TGFβ signaling contributes to the fibrotic remodeling of the mutant GFs.

**Figure 9:**
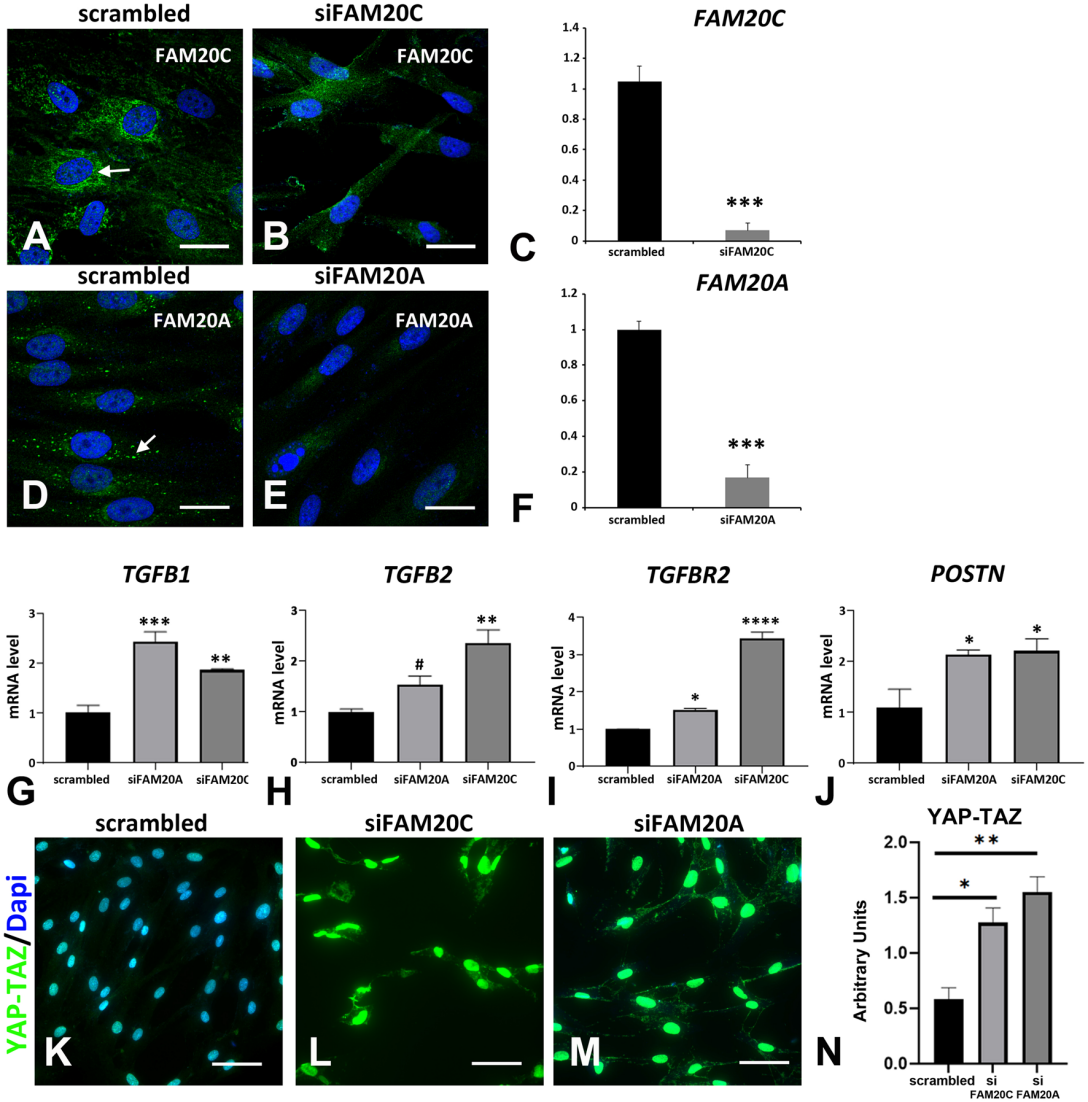
*FAM20C* and *FAM20A* silencing. (**A**–**G**) GFs were transfected with siRNAs targeted either *FAM20C*, *FAM20A* or scrambled siRNAs used as controls. Three days after transfection, cells were analyzed for FAM20C or FAM20A expression using immunocytochemistry (**A**,**B**,**D**,**E**) or QPCR (**C**,**F**). (**A**) FAM20C protein was localized to endoplasmic reticulum in GFs transfected with scrambled siRNAs. (**B**) This staining was not observed in GFs transfected with siRNAs targeted *FAM20C*. (**C**) Using real time RT-PCR, a 90% decrease in mRNA to *FAM20C* was observed. (**D**) FAM20A protein was localized in large vesicles in GFs transfected with scrambled siRNAs. (**E**) This staining was absent in GFs transfected with siRNAs targeting *FAM20A*. (**F**) Using real time RT-PCR, a significant 80% decrease in mRNA to *FAM20A* was observed. (**G**–**J**) Real Time RT-PCR analysis of candidate genes corresponding to *TGFB1* (**G**), *TGFB2* (**H**), the receptor *TGFBR2* (**I**) and Periostin *POSTN* (**J**). Scrambled siRNAs, si*FAM20C* or si*FAM20A* values correspond to the mean of 3 independent experiments in triplicates of three GFs cultures. Datas represent mean fold gene expressions ± s.d. Data was analyzed via one-way ANOVA with Bonferroni multiple comparisons test (*p < 0.05, **p < 0.01, ***p < 0.005, ****p < 0.001). (**K**–**M**) YAP-TAZ immunocytochemistry showed some lightly immunoreactive nuclei in GFs transfected with scrambled siRNAs (**K**). In GFs transfected with si*FAM20C* or si*FAM20A* a dramatic increase in the staining was observed in all nuclei (**L**,**M**). (**N**) Graph representation of the relative fluorescence intensity of YAP-TAZ in GFs transfected with scrambled siRNAs, si*FAM20C* or si*FAM20A*. Statistically significant differences are shown as ***P < 0.001. Scale bars: (**A**,**B**,**D**,**E**) = 20 µm; (**K**–**M**) = 50 µm.

To confirm the interaction loop between FAM20C and TGFβ actors and targets we used a siRNA approach on primary human GFs to deplete FAM20C. We used the same approach to comparatively study the impact of FAM20A depletion. The knockdown was efficient for both *FAM20C* and *FAM20A* (Fig. 9A–F). Compared with the control siRNA the *FAM20C*- and *FAM20A*-depleted cells had a more elongated morphology (Fig. 9B,E). Both *FAM20C* and *FAM20A* siRNAs but not the control one resulted in a strong upregulation of TGFB1 (Fig. 9G). *TGFB2* was significantly upregulated in *FAM20C*-depleted cells (Fig. 9H) whereas *TGFBR2* was strongly upregulated in both (Fig. 9I). Consistent with TGFβ signaling activation the depletion of *FAM20C* or *FAM20A* led to a significant upregulation of the established TGFβ target and fibrotic remodeling marker POSTN (Fig. 9J). We further investigated whether the YAP/TAZ pathway was activated in the knockdown cells. Immunostaining revealed a predominantly nuclear localization of YAP/TAZ in both *FAM20C*- and *FAM20A*-depleted GFs (Fig. 9K–N and Supplementary Fig. 8) confirming the activation of YAP/TAZ signaling.

The above data clearly showed that dysfunction or knockdown of FAM20C similarly activated the TGFβ pathway, the primary mediator of the fibrogenesis process. Our results also uncovered the contribution of YAP/TAZ in FAM20C-associated gingival fibrosis confirming published data on the YAP/TAZ-TGFβ cross-talk^55^.

## Discussion

In this study we characterized the gingival phenotype of RNS patients and showed for the first time that FAM20C dysfunction impaired TGFβ and YAP/TAZ signaling pathways in the fibrotic RNS gingiva.

Using proteomic and biochemical approaches we found that the major dysregulated proteins in RNS-GFs were associated with ECM, actin filament and collagen fibril organization as well as bone development and ossification. The immunomorphological in vivo and in vitro data confirmed the fibrotic and osteogenic potential of the RNS-GFs, showed that the TGFβ cascade was strongly activated in these cells and revealed that TGFβ hyperactivation was associated with increased YAP/TAZ signaling. Subsequent FAM20C depletion from control cells resulted in a similar activation of the TGFβ-YAP/TAZ pathway, confirming the interaction of FAM20C with the profibrotic TGFβ-YAP/TAZ network.

FAM20C is a ubiquitous protein kinase phosphorylating numerous secreted proteins including the ones involved in calcium binding and biomineralization. Pathogenic variants that abolish the kinase activity result in a usually perinatal lethality whereas partial activity is compatible with life. RNS-1 and RNS-2 patients were previously described^32^. The homozygous missense variant in RNS-1 was predicted to result in a residual kinase activity whereas in the homozygous splice site mutation in RNS-2 some wild-type sized *FAM20C* transcript was detected and the clinical phenotype was milder. In this study, the histopathological analysis showed that compared to RNS-2, the RNS-1 gingival connective tissue appeared more disorganized, much more vascularized and contained less calcified deposits indicating that the gingival modifications were to some extent variant-specific. The in vitro RNS-GFs data were consistent with the genetic, clinical and histological variability, they also suggested that the specific FAM20C mutations could result in altered distribution and/or processing and/or expression level of the mutant proteins. We did not identify significantly modified FAM20C mRNA levels in the patients’ GFs. This piece of result is consistent with the nature of the variants and resulting proteins, i.e. the P496L substitution (RNS-1) is thought to decrease the kinase activity of the mutant protein but not protein synthesis whereas in the RNS-2 patient normal protein synthesis albeit at lower levels, is expected to occur. In terms of protein distribution however, both mutant forms of FAM20C abnormally accumulated in the ER. A similar distribution has previously been reported in HEK293T cells overexpressing mutant FAM20C constructs^31^; to our knowledge this is the first time that a similar result is reported for native RNS-derived GFs.

FAM20C is a Golgi-localized secretory kinase and its activity is optimal in the Golgi milieu. Nevertheless, it is possible that FAM20C is active during its transit across the ER and several ER/Golgi localized proteins are FAM20C targets. Ero1α, as well as proteins involved in intracellular calcium storage handling, including the histidine rich calcium binding protein (HRC), calreticulin and calsequestrin-2 are phosphorylated by FAM20C^24,61,62^. The role of FAM20C in ER protein phosphorylation is further supported by the observation that STIM1, mainly localized in the ER is a FAM20C target^24^. The mutant forms of FAM20C described here are thought to retain some kinase activity^32^. A prolonged residence of mutant FAM20C protein in the ER and aberrant phosphorylation of its local targets could thus modify their function, disrupt calcium regulation and contribute to ER stress. In addition, ER accumulation of FAM20C would prevent the S1P-mediated proteolysis of FAM20C in the Golgi and block further secretion and autophosphorylation^63^. Although the significance of the last modification for FAM20C activity is unclear, blocking the secretion could impair biomineralization processes and favor ectopic calcification^63^.

Because of the broad substrate repertoire of FAM20C it is difficult to define singular events dictating FAM20C functions. However, the role of FAM20C in ER calcium storage regulation and protein secretion strongly suggests that FAM20C may be broadly regulating ER calcium associated processes and ER stress^64^. ER stress has previously been described in cells overexpressing mutant FAM20C forms^65^. Our data showing subtle differences in cytosolic and nuclear distribution of UCHL1 in RNS-GFs may suggest impaired protein turnover^66^ but additional experiments are required to test this hypothesis.

ER stress favors the release of pro-fibrotic factors, the activation of molecular pathways such as TGFβ signaling and contributes to the pathogenesis of idiopathic pulmonary, renal and cardiac fibrosis^67–69^. Our observations clearly support the idea that FAM20C modulates TGFβ signaling: we showed that FAM20C dysfunction or silencing similarly resulted in hyperactivation of the TGFβ pathway. Our data thus indicate that FAM20C is required for normal TGFβ activity and GF homeostasis, indirectly preventing fibrotic remodeling and ectopic calcifications.

Protein kinases including FAM20C, phosphorylate numerous functionally distinct proteins, changing thus the activity of the substrate, mediating transcriptional regulation, and modulating protein secretion in health and disease^45,70^. Several groups have indeed shown that FAM20C is essential not only in ER homeostasis but also in coagulation, hormonal regulation, cardiac health and biomineralization. We here broaden the spectrum of FAM20C target tissues and show that gingival health also requires intact FAM20C activity.

In vitro, mineralization-inducing conditions resulted in the formation of calcium deposits in RNS-GFs. This result was compatible with the reported human phenotype but contrasted with murine in vitro and in vivo studies using *Fam20C* KO mice or cells^19,30,32,36,39,71^. Ectopic calcification was observed exclusively in the eye of the *Fam20C* null mutant^39^ and the formation of mineralized nodules was low in murine Fam20C-deficient osteoblasts^19^. This discrepancy can be explained by the histological and anatomical differences between human and murine gingiva, the different cell types used and the presence or not of a residual FAM20C activity. We previously showed that human GFs expressed minute amounts of osteogenic genes and this expression remained low even under mineralization-inducing conditions^8^. Consistent with the previous and current data the formation of calcified nodules was very limited in the control GFs but more pronounced in RNS-GFs implicating FAM20C in aberrant soft tissue mineralization.

Fibrosis and ectopic mineralization were intertwined in the RNS gingiva. The pathogenesis of these processes was highlighted by the biochemical analyses indicating excessive ECM protein production together with dysregulated matrix remodeling.

Myofibroblasts, generally α-SMA positive^72–74^, are essential in fibrotic processes and absent from normal gingiva. Myofibroblasts synthesize most of the structural and matricellular ECM proteins and contribute to matrix remodeling by producing various proteases including MMPs and their inhibitors TIMPs^75–78^. We found that the expression of α-SMA, was strongly increased in RNS-GFs and this was correlated with increased levels of IL-6, known to promote differentiation of fibroblasts into myofibroblasts and trigger collagen synthesis^79^. Enhanced IL-6 production was indeed previously associated with gingival overgrowth^80^. The expression of POSTN and VIM thought to be myofibroblast markers in multiple tissues^81,82^ was also particularly high and was accompanied by the abundantly expressed type I collagen and the matricellular proteins SPARC and TSP2, all involved in fibrosis progression including in drug-induced gingival fibrosis^83–88^.

In addition to the above-described excessive ECM production, matrix remodeling was defective in RNS-GFs. High levels of the serine protease inhibitor SERPINE1, generally observed in fibrotic tissues^89^ and of MMP3 involved in the degradation of laminins and other basement membrane proteins^90^ could exaggerate the accumulation of ECM and fibrosis. Downregulation of the collagenase MMP1, specific for collagens I–III, and its main inhibitor TIMP1 could further perturb gingival tissue remodeling. Decreased MMP1, but not TIMP1, production was indeed previously associated with hereditary gingival fibromatosis (HGF), a genetically heterogeneous condition characterized by pathological fibrosis of gingival tissue and abundant production of type I collagen and VIM^91^. FN1 regulates matrix metalloproteinase expression and was functionally associated with gingival overgrowth in HGF^92^. Low amounts of FN1 were rather surprising but could be attributed to increased protein degradation including by MMP3^93^. The same could stand for the discrepancy between FNDC1 protein and transcript levels. FNDC1 and FN1 are functionally and structurally close and thought to be coregulated; indeed, FNDC1-dependent FN1 expression was observed in prostate cancer^94,95^. It is currently unknown whether a similar regulatory pathway operates in RNS-GFs but the decrease in FNDC1 protein could be compatible with perturbed ossification^96^ and the rather low abundance of calcified nodules in RNS gingiva.

It is unclear why for some proteins including the above mentioned FNDC1 and MMP3 the mRNA and protein levels are discordant. In view of the regulatory role of FAM20C on protein secretion^45^ we propose that increased secretion of MMP3 despite normal synthesis, or intracellular retention of FNDC1 despite normal (RNS1) or increased (RNS2) levels may explain these results. Alternatively, as indicated above, low protein levels may result from increased protein degradation.

Our findings, i.e. increased ECM protein synthesis, a shift in the protease/antiprotease balance towards a matrix-preserving process and increased production of TGF-regulated factors suggested that this pathway played a key role in RNS gingival fibrosis. The central role of TGFβ in fibrotic disease is established including in hereditary and acquired gingival fibromatosis^49,51,97,98^. We indeed recently showed that this pathway was hyperactivated in ERS GFs and tissues expressing mutant forms of FAM20A^42^.

We here showed that a similar hyperactivation occurs in RNS-derived gingival tissue and cells. Our present results revealed that Smad3, the crucial TGFβ-signaling mediator in fibrosis was intrinsically activated/phosphorylated in RNS gingiva and GFs, which synthesized excessive amounts of collagen I-associated ECM. The levels of *TGFB1* mRNA were strongly increased in the RNS GFs; a similar upregulation was previously associated with the activation of YAP/TAZ signaling in the dermis^99^. Nuclear YAP/TAZ promotes TGFβ signaling via retaining activated Smad3 in the nucleus^100^. The level of YAP/TAZ was higher in RNS Gfs compared to control GFs and the amount of the inactive phospho-YAP dramatically decreased suggesting a shift towards YAP/TAZ activation. Addition of TGFβ1 further increased active YAP/TAZ levels consistent with converging TGFβ and YAP/TAZ signaling in RNS GFs. We investigated the FAM20C/TGFβ/YAP/TAZ link by knocking down *FAM20C* and indeed showed that silencing of *FAM20C* was sufficient to promote *POSTN* upregulation and nuclear YAP/TAZ localization. Nuclear YAP/TAZ localization was also observed in the control cells. It is very likely that the culture conditions, i.e. cells grown on a hard substrate like plastic, were sufficient to trigger YAP/TAZ nuclear translocation^101^. It is beyond the scope of this work to detail the mechanical regulation of YAP/TAZ expression; we assume that the mechanical stress information caused by the culture conditions was similar in control and mutant cells. Nevertheless, the nuclear YAP/TAZ signal was much stronger after *FAM20C* silencing suggesting a stronger activation in the depleted cells and providing a direct link between FAM20C function and TGFβ/YAP/TAZ signaling. It is very interesting to note that we obtained the same results by knocking down *FAM20A*. The interdependence between FAM20A and FAM20C concerning the kinase activity of the latter has been described^16^. Our finding that both FAM20C and FAM20A may be involved in the same signaling pathways suggests additional, yet to be uncovered cellular functions of these proteins.

Published data on FAM20C and FAM20A dysfunction in humans and mice^19,102–104^ described increased and/or attenuated BMP signaling in ectopic intrapulpal calcifications, AI, root defects and salivary gland development. Our previous results, in ERS GFs, and present proteomic analyses did not show any modification in BMP actors indicating that this pathway may not be directly involved in the fibrotic/osteogenic modifications of the gingiva.

In summary, our in vivo and in vitro data provide a novel and detailed description of the RNS gingival phenotype. We show that excessive ECM production and impaired matrix remodeling are the imperatives for gingival fibrosis and calcification in RNS patients. Our finding that both TGFβ and YAP/TAZ signaling are activated is completely new and highlights new aspects of FAM20C activity in signaling regulation.

## Methods

### Ethics—patients recruitment

Patients were referred for oral rehabilitation at the Reference Center of rare dental diseases (Oral Center for Inherited Diseases, University Hospital of Brasília, Brasil). Diagnosis of RNS was based on clinical and radiological features^5,8^. Patients and healthy age-matched controls (n = 3) were recruited following informed consent in accordance with the principles outlined in the declaration of Helsinki. Written informed consent was obtained from probands for the publication of any potentially identifiable images or data included in this article. This project was approved by the Research Ethics Committee of the Faculty of Medicine of the University of Brasília (CAAE: 34149814.1.0000.5558). Samples from probands and controls were harvested during oral rehabilitation and were prepared for histological or cell culture analyses (authorization CODECOH DC-2018-3382).

### Immunocytochemistry and histology

Approximately, 1 cm^3^ gingival samples from patients were fixed for 24 h at 4 °C in 4% paraformaldehyde and then embedded in paraffin wax.

#### Histology

Sections were stained with routine protocols for Haematoxylin and Eosin (HE), Picro-Sirius Red, and Alizarin Red stains.

#### Immunohistochemistry

Epitope retrieval was achieved by heat. Sections were incubated for 2 h at room temperature or overnight at 4 °C with primary antibodies; rabbit anti-collagen V alpha-1 (1/1000; NBP-38162; Novus biologicals), rabbit anti-FAM20A (1/250; OACD03385; Aviva), rabbit anti-Fam20C (1/250; OAAB01003; Aviva), rabbit anti-Periostin (1/500; ab14041; Abcam), rabbit anti-phospho-SMAD3 (1/500; ab52903; Abcam), rabbit anti-SPARC (1/2000; ab255733; ABCAM), mouse anti-alpha Smooth Muscle Actin (1/1000; 1A4; ab7817; Abcam), mouse anti-Fibronectin (1/1000; P1H11; DSHB), mouse anti-FSP1 (1/2000; ab218512; ABCAM), mouse anti-Vimentin (1/1000; RV203; NBP-19767; Novus biologicals) and rat anti-Procollagen type I (1/500; M-58; ab64409; Abcam), mouse anti-Ubiquitin (UCHL1; 1/400; 66230-1-Ig; Proteintech), goat anti-Lamin B1(1/200; sc6216), Concanavalin A-Alexa 633 conjugate (70 mg/ml; 2616059; Thermo Fischer). Secondary antibodies used were Alexa 488- or Cy3-conjugated donkey anti-rabbit (Jackson Immunoresearch Laboratories, West Grove, PA; 1:500), Alexa 488- or Cy3-conjugated donkey anti-mouse (Invitrogen, Eugene, OR; 1:500), and Alexa 488- or Cy3-conjugated donkey anti-goat (1:500). Nuclear staining was achieved by 20 min incubation at room temperature in Hoechst 33,342 (Invitrogen, Eugene, OR). Images were collected by confocal microscopy (Zeiss LSM8) and processed using ZEN version 3,8(Carl Zeiss Microscopy GmbH, Jena, Germany) and ImageJ software (http://rsweb.nih.gov/ij/index.html).

DAB (3,3’-Diaminobenzidine) staining was achieved using standard protocol (donkey anti-rabbit biotin conjugated, ab7082; streptavidin-HRP, ab64269; DAB substrate kit, ab64238; all products, Abcam).

### Fibroblast cell culture

Controls and proband gingival fibroblasts were established by plating small pieces of excised gingival on plastic dishes. Cells, particularly gingival fibroblasts, migrate out of the explant and colonize the petri dish. The flasks were filled with Dulbecco’s modified Eagle’s medium–low glucose (DMEM) containing 20% fetal calf serum (FCS), 1% non-essential amino acid, penicillin/streptomycin (100 mg/mL) and amphotericin B (2 ng/mL). The flasks are then placed in an incubator programmed at 37 °C in a humidity atmosphere at 5% CO_2_ and the cell culture medium was changed twice a week until the confluence of the cells (90% after about 3 weeks). Once at confluence, the gingival fibroblasts were trypsinized (Trypsin–EDTA, GIBCO^®^, 1 mL at 0.05%) and single-cell suspensions were seeded in 25 cm^2^ flasks containing DMEM 10% of FCS, passaged by splitting when they reached confluence, and frozen in liquid nitrogen until use. Cells at passages 3 to 6 were used in all experiments. We checked each cell culture for the morphology and the marker of fibroblasts (fibroblast-specific protein 1 [FSP1]; ab27957; Abcam, Cambridge, UK). We confirmed that all cell cultures did not exhibit the morphological changes during the passages, and that FSP1 was clearly detected in these cells (data not shown). Each experiment using these cells was repeated at least three times.

For the experimentations, GFs were seeded and cultured in DMEM 10% FBS for three days and then cultured in a BSA/FBS free medium for two days. These conditioned supernatants were used to perform the secretome analysis. Cultured supernatants were analysed by LC–MS/MS. The remaining cell lysates were used to the proteome analysis. Using the culture conditions, cells were collected for RT-qPCR and Western blot analyses. Cells were seeded at 7000 cells per cm^2^ surface area in 6 well plates for RT-qPCR and in dishes (100 mm diameter) for western blot experiments.

### TGF-β1 treatment

For TGF-β1 treatment, recombinant human TGF-β1 at 5 ng/ml was used (R&D Systems, MN, USA). Prior to TGF-β1 treatment, nearly confluent cells were serum-starved in low-glucose DMEM for 24 h and washed with serum-free DMEM. Immediately after, cells were treated with TGF-β1 for 6 h.

For RT-qPCR and Western blot analyses cells were seeded at 7000 cells per cm^2^ surface area in 6 well plates and in 100 mm diameter dishes respectively. For immunofluorescence cells chamber slides (Nunc Lab-Tek, Thermofisher) were used. GFs from two controls and RNS subjects were used for all the experiments. Three replicates of each experiment were performed for each test to ensure reproducibility.

### Si RNA transfection

The loss of function analysis for *FAM20A* and *FAM20C* was performed using small interfering RNA (siRNA). Three unique siRNAs *per* gene (Flexitube siRNA, Hs_FAM20A_3, Hs_FAM20C_3; Qiagen) were used along with a control scrambled siRNA (AllStars Negative Control siRNA, Qiagen).

Gingival fibroblasts, exhibiting a confluence of 50–70%, were pretreated with 10% FCS (fetal calf serum) supplemented DMEM (Dulbecco’s Modified Eagle Medium) without antibiotics. Prior to transfection siRNAs were complexed with Lipofectamine^TM^ RNAi REAGENT (InVitroGen). Gingival fibroblasts were transfected for 6 h following the protocol outlined by Thermo Fisher Scientific. The subsequent incubation was carried out at 37 °C with a 5% CO_2_ environment for a period ranging from 1 to 3 days.

For RT-qPCR cells were seeded at 7000 cells per cm^2^ surface area in 6 well plates. For immunofluorescence cells chamber slides (Nunc Lab-Tek, Thermofisher) were used. Three replicates of each experiment were performed for each test to ensure reproducibility.

### Mass spectrometry

A high-resolution mass spectrometry (MS)-based approach was used to perform differential quantitative analysis on both secreted and cell lysate proteins from GFs cultures. Independent GFs cultures (control and RS) were seeded and cultured in triplicates, in low glucose DMEM 10% FBS for three days and then serum-deprived DMEM for two additional days. Each serum free cell supernatant (Control, n = 3; RNS-1, n = 3; RNS-2, n = 3) and remaining cell lysate (Control, n = 3; RNS-1, n = 3; RNS-2, n = 3) was analyzed in a single-run of LC–MS/MS.

### Proteomic analysis

#### Sample preparation for proteomic mass spectrometry

Samples were prepared as previously described^42^. Sample preparation was different for secretome or cell lysate. For secretome analysis, each sample was precipitated with DOC/TCA (0.1%/10%) to concentrate the protein pool. After precipitation, proteins were resuspended in a homemade solubilization buffer. For cell proteome analysis, cell lysate was harvested using FastPrep Technologies (MP Biomedicals) and resuspended in a solubilization buffer. Then protein concentration of all samples was estimated using Bradford Assay (Biorad), according to the manufacturer’s instructions. Based on Bradford results, 25 µg of proteins of each sample were loaded into a 7% polyacrylamide gel (Acrylamide/Bis-Acrylamide 30% [29:1], Sigma Aldrich) and a migration was performed in a short period (90 min at 10–20 mA/gel) to stack all proteins in in a small piece of gel. After Coomassie blue staining, the revealed protein bands were excised. Proteins were reduced with 5 mM dithiothreitol for 40 min followed by alkylation with 20 mM iodoacetamide for 40 min in the dark (all products from Sigma Aldrich). After washing steps with water and acetonitrile (Sigma Aldrich), gel bands were submitted to protein digestion by 1 µg of trypsin (Promega). After overnight incubation at 37 °C, several steps of peptide extraction were performed with of 0.1% formic acid (FA) in water and acetonitrile solutions. Finally, for each sample, peptide fractions were combined and dried.

### Nano LC–MS/MS analysis

For each sample, peptide fractions were solubilized in FA 0.1% (v/v) and analyzed on a LTQ-Orbitrap Elite apparatus coupled to an Easy nanoLC II system (Thermo Scientific). An amount of 0.2 µg of peptides was injected onto an enrichment column (C18 Pepmap100, Thermo Scientific). The separation was carried out with an analytical column needle (NTCC-360/100-5-153, Nikkyo-Technos). The flow rate was 300 nL/min and the mobile phase composed of H_2_O/0.1% FA (buffer A) and ACN/0.1% FA (buffer B). The elution gradient duration was 120 min: 0-106 min, 2–40% B; 106-110 min, 40–100% B; 110-120 min, 100% B. The mass spectrometer was operated in positive mode with CID fragmentation. For mass spectrometry settings, the capillary voltage was 1.5 kV and the temperature of the capillary was 275 °C. The *m*/*z* detection range was 400–1800 in MS scan at a resolution of 60 000. The 20 most intense peptide ions were selected and the fragmentation occurred with a normalized collision energy of 35. Dynamic exclusion of already fragmented precursor ions was applied for 30 s.

### Quantification and statistical analysis

After MS analysis, raw data were imported into Progenesis QI v3.0 software(waters corporation,nonlinear dynamics, (https://www.nonlinear.com/progenesis/qi/v3.0/download/). To perform quantification, the peptide maps alignment and normalization had to be done. Briefly, one sample was set as a reference to align retention times of all other samples and a normalization step managed by the software (based on the idea that much of the peptides remain with unmodified abundances between the experiments, and so the quantitative abundance ratio of these majority of features should equal to 1), allowed us to determine a scalar factor for each sample, thereafter used for normalization. Statistical analysis was performed using the in-built *Progenesis* statistical box ‘one-way ANOVA’. MS/MS spectra were then exported for peptide identification with Mascot (Matrix Science, version 2.6.0). Database searches were performed with the following parameters: taxonomy: human (22,244 sequences); 1 missed cleavage; variable modification: carbamidomethyl of cysteine and oxidation of methionine. Mass tolerances for precursor and fragment ions were 10 ppm and 0.35 Da respectively. False discovery rates were calculated using a decoy-fusion approach in Mascot. Identified spectrum matches with -10logP value of 20 or higher were kept, at a FDR threshold of 5%. Mascot search results were imported into *Progenesis*. For each condition, the total cumulative abundance of protein was calculated by summing the abundance of peptides. All proteins identified with at least 2 peptides were considered for further analysis. A protein was considered differentially expressed if the *p* value is inferior to 0.05 and the statistical value of Power superior to 0.8.

### Network biology and systems level analysis

Proteins identified by MS were entered in STRING database (https://string-db.org/) to create a protein–protein association network^105^. Network nodes represent all the proteins produced by a single protein-coding gene locus noting that splice isoforms or post-translational modifications are collapsed. Edges represent protein–protein associations that are specific and meaningful, such as proteins that jointly contribute to a shared function (note that this does not necessarily mean they physically bind each other). Thickness of network edges indicates the strength of data support based on textmining, experiments, databases, co-expression, neighbourhood, gene fusion and co-occurrence. The minimum required interaction score was set to a high confidence level of 0.7. Predicted interactome was evaluated using String software based on annotation enrichment strategy. The process of String to attach biological information relied on a series of enrichment-based tools widely used for the analysis of genes that are differentially expressed including Gene Ontology Resource (GO; geneontology.org), PANTHER16.0 (pantherdb.org), KEGG, or DAVID.

### Western blotting

Western blotting was performed as previously described^8^. In brief, GFs were washed twice with PBS and protein was isolated in RIPA buffer (Sigma Aldrich) containing protease (Roche Diagnostics) and phosphatase inhibitors (Calbiochem) cocktails. Protein concentration was determined by Pierce^®^ BCA Protein assay kit (Pierce; Waltham, MA, USA). A 25 µg quantity of protein from each sample was separated by sodium dodecyl sulphate polyacrylamide gel electrophoresis (SDS-PAGE) and transferred to nitrocellulose membranes. Membranes were washed with Tris-buffered saline containing 0.05% Tween-20 (TBS-T) and blocked with 5% dried milk in TBS-T. Primary antibodies for anti-alpha Smooth Muscle Actin (1/1000; 14,968; Cell Signaling Technologies), phospho-YAP1Ser397 (1/2,000; 29018-1-AP; Proteintech), YAP-TAZ (1/1000; 93,622; Cell Signaling Technologies) and GAPDH (MAB374; Millipore; 1:2000) were used to incubate the membranes for 12 h. Detection was with appropriate peroxidase-conjugated secondary antibodies (Jackson ImmunoResearch; West Grove, PA, USA; 1:2000), which were developed with SuperSignal Western Pico Chemiluminescence Substrate (Pierce).

### Quantitative RT-qPCR

Total RNA was isolated using commercially available kits according to manufacturer guidelines (RNeasy Mini, Qiagen) and measured (Nanodrop, Peqleb). One μg was used in a reverse transcription reaction (SuperScript First strand synthesis, Thermofisher). Quantitative-PCR was performed using Quantifast SYBR Green PCR Kit (Qiagen), reactions were performed in triplicate. Transcript levels were calculated using the standard curves generated using serial dilutions of cDNA obtained by reverse transcription of control RNA samples then normalized to HPRT. Primer sequences were listed in supplementary Table S15. Amplification specificities were assessed by melting curve analyses and amplicons were sequenced. Values correspond to the mean of 3 independent experiments in triplicates of three control cultures and the RNS patient cultures. Data represent mean fold gene expressions ± s.d. relative to control. Data were analyzed via two-way ANOVA with Bonferroni multiple comparisons test (*p < 0.05, **p < 0.01, ***p < 0.001).

### Immunocytochemistry in vitro

Cells were fixed with 4% paraformaldehyde, permeabilized with 0.1% Triton X-100, and blocked with 1% bovine serum albumin. Primary antibodies for phospho-SMAD3 (1/500; ab52903; Abcam) and YAP-TAZ (1/1000; 93,622; Cell Signaling Technologies) were incubated one hour. Phalloidin staining was achieved simultaneously (Alexa 594 Phalloidin, A12381, ThermoFisher Scientific) Secondary antibodies used were Alexa 488- donkey anti-rabbit (Jackson Immunoresearch Laboratories, West Grove, PA; 1:500). Nuclear staining was achieved by 20 min incubation at room temperature in Hoechst 33,342 (ThermoFisher Scientific). No cellular autofluorescence and no nonspecific labeling were detected in these conditions. Images were collected by confocal microscopy (Zeiss LSM8) and processed using ZEN (Zeiss) and ImageJ software.

### Image acquisition

Images were obtained on a Zeiss LSM800 confocal microscope (Carl Zeiss Microscopy). In the only step involving operator discretion, all image parameters including pinhole size, detector gain, amplifier offset, amplifier gain and laser intensity were first set for the DAPI and specific-antigen channels using normal control tissue, and the same setting used for all samples imaged on a given day. Frame size, scan speed and averaging were the same for all images. For each sample four non-overlapping images for each channel were acquired and stored as 16-bit fluorescent images (TIFF) for analysis.

### Image analysis

Image analysis was performed in the ImageJ software program.

#### Fluorescence

The original DAPI and specific antigen images are opened; an intensity threshold for the DAPI signal is chosen by the user. Pixels with signals above this threshold constitute DAPI regions of interest (ROI). ROI are converted into a binary mask where areas of interest have a pixel intensity value of 1 and background has a pixel intensity value of 0. In order to eliminate background noise, ROI areas smaller than 3 × 3 contiguous pixels are removed from the image by an erosion step, leaving areas of equal to or greater than 3 × 3 contiguous pixels that are presumed to define contiguous nuclear membrane. The resulting image is dilated to restore positive regions. The dilated image is used to create a DAPI mask. The DAPI mask is overlaid on to the DAPI image and intensities are quantified. The DAPI mask is overlaid onto the specific-antigen image and intensities are quantified. The Excel file contains values for average specific-antigen intensity, average DAPI intensity, and a specifc-antigen/DAPI ratio for each image pair analysed. Average specific-antigen intensity was divided by average DAPI intensity to give a specific-antigen/DAPI ratio. These values were recorded for each of the four images per sample. The four specific-antigen/DAPI ratio values were themselves averaged to generate an overall specific-antigen/DAPI ratio for each sample.

#### DAB

In brief, for color deconvolution of IHC images, DAB and hematoxylin staining were digitally separated using ImageJ Fiji software (https://imagej.net/software/fiji/) with a color deconvolution plugin. Deconvoluted images with DAB staining were subjected to measurement of mean gray values, with the lower and upper thresholds set at 120 and 220 for FAM20C and FAM20A. The number of nuclei in the same field was determined from the hematoxylin staining by analyzing particle numbers using ImageJ Fiji software. The mean gray values of FAM20C and FAM20A were then normalized by the number of cell nuclei. The expression levels of FAM20C and FAM20A were determined as the average of the gray values normalized by nuclei number from 4 images per gingival tissue determined.

### Statistical analysis

Statistical analysis was by one-way or two-way ANOVA, as appropriate, followed by Bonferroni multiple comparisons test with Graphpad Software version 5 (Graphpad Software; La Jolla, CA, USA) (https://www.graphpad.com/support/prism-5-updates/) (p < 0.05 was considered significant). Data are expressed as the mean ± standard deviation of 3 or 5 individual experiments with independent primary cultures from different subjects. Individual experiments included three replicates.

### Institutional review board statement

This project was approved by the Research Ethics Committee of the Faculty of Medicine of the University of Brasília (CAAE: 34149814.1.0000.5558). The studies involving human gingival fibroblast cultures were reviewed and approved by CODECOH DC-2018-3382 INSERM.

### Informed consent statement

Written informed consent to participate in this study was provided by the participants’ legal guardian/next of kin.

### Supplementary Information

Supplementary Figures.

Supplementary Legends.

Supplementary Table S1.

Supplementary Table S2.

Supplementary Table S3.

Supplementary Table S4.

Supplementary Table S5.

Supplementary Table S6.

Supplementary Table S7.

Supplementary Table S8.

Supplementary Table S9.

Supplementary Table S10.

Supplementary Table S11.

Supplementary Table S12.

Supplementary Table S13.

Supplementary Table S14.

Supplementary Table S15.

## Data Availability

The proteomic datasets used during the current study available from the corresponding author on reasonable request. All data generated during this study are included in this published article [and its supplementary information files].
