# Supplementary material for: Gingival proteomics reveals the role of TGF beta and YAP/TAZ signaling in Raine syndrome fibrosis
Source: Sci Rep. 2024 Apr 25;14:9497. doi: 10.1038/s41598-024-59713-0 (PMC11045870; doi:10.1038/s41598-024-59713-0)
Supplement: Supplementary file 1 — Supplementary Figures. [file 41598_2024_59713_MOESM1_ESM.pdf]

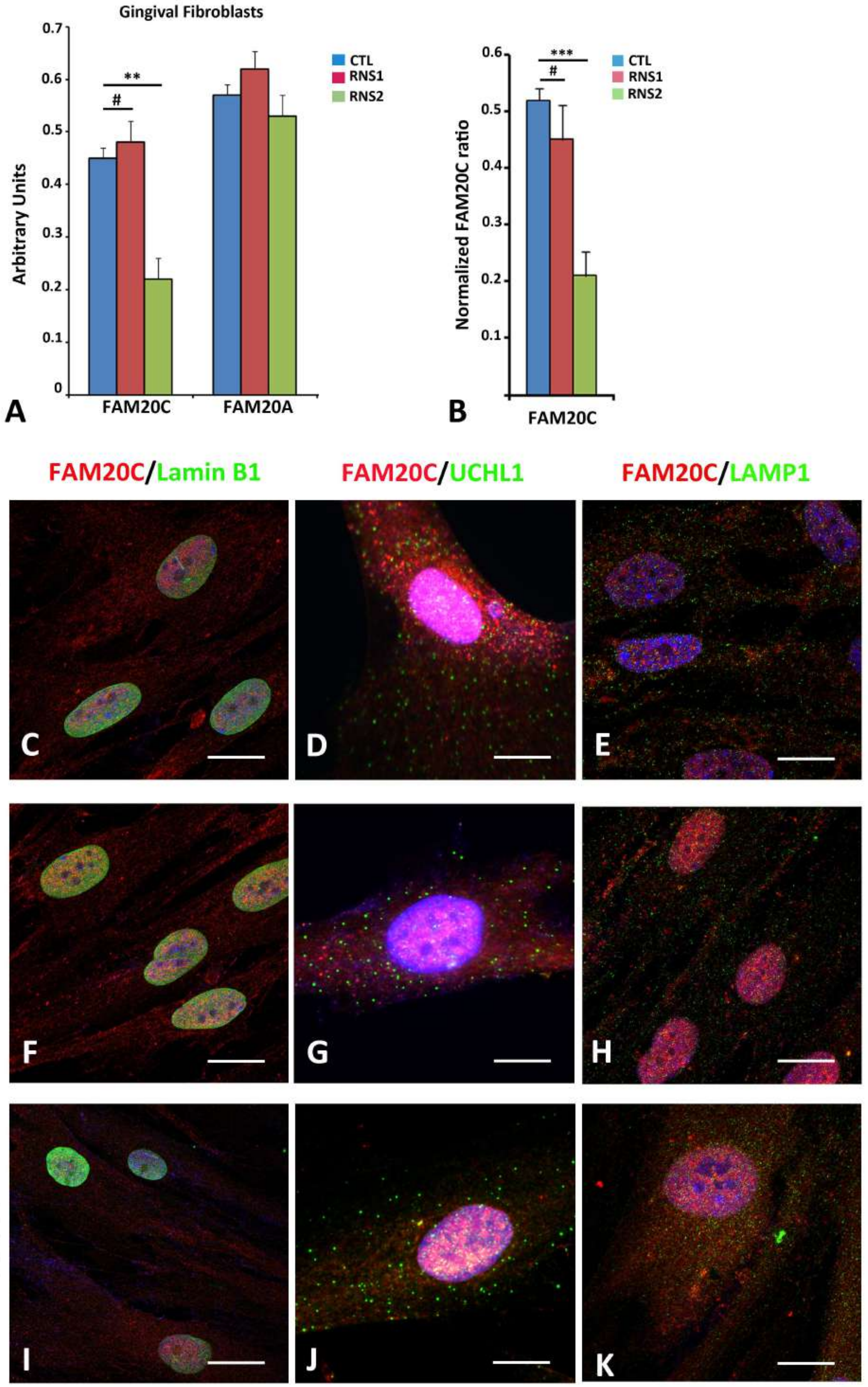

Supplementary figure 1: (A) Immunohistochemical quantification of FAM20C and FAM20A on gingival sections from control (CTL), RNS-1 or RNS-2 specimens. Graph representation of the DAB quantification of FAM20C and FAM20A. Statistically significant differences are shown as ***P<0.001. (B) Quantitative analysis of fluorescence staining of FAM20C in gingival fibroblast in vitro. Graph representation of the relative fluorescence intensity of FAM20C in gingival fibroblasts from control, RNS-1 and RNS-2 cultures. Statistically significant differences are shon as ***P<0.001. (C, F, I) Fluorescencelocalization of FAM20C gren) and the nuclear specific marker, Lamin B1 (red) in normal(C), RNS-1 (F) and RNS-2 (I) GFs. (D, G, J) Fluorescence localization of FAM20C (green) and the specific protein degradation marker, ubiquitin C-terminal hydrolase L1 (UCHL1; red) in normal (D), RNS-1 (G) and RNS-2 (J) GFs. 5E, H, K) Fluorescence localization of FAM20c (green) and the lysosomal protein, Lamp1 (red) in normal (E), RNS-1 (H), RNS-2 (K) Gfs. Scale bars: C, F, I, E, H, K = 40 μm; D, G, J = 25 μm.

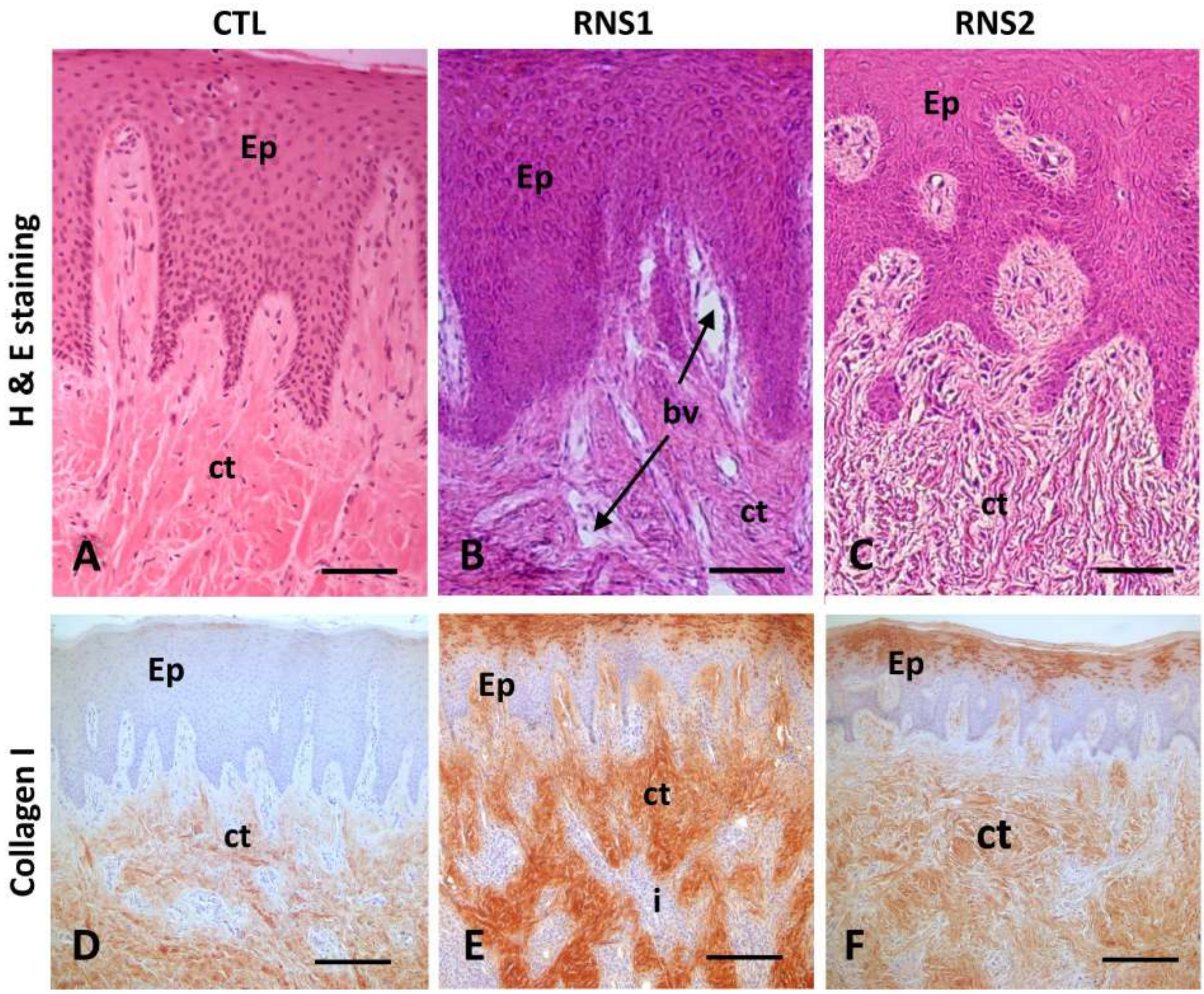

Supplementary Figure 2: Histologic features of RNS mutants. (**A-C**) Hematoxylin-eosin staining shows numerous tortuous blood vessels (bv) with an abnormally large diameter in the papillary layer and associated extensive inflammatory infiltrates in RNS-1 gingiva (**B**). Abnormally large and shredded collagen bundles running in all directions are found in RNS-2 gingiva (**C**). (**D-F**) Collagen I immunostaining showed an increase of collagen I in the connective tissue of RNS-1 and RNS-2 mutant gingivas. (**D**) Fibers of collagen organize in bundles of fixed diameter running in perpendicular pathways. In RNS-1 (**E**) and RNS-2 (**F**), fibers do not organize in packed bundles, but appeared shredded. Scale bars: A-C = 200 µm; D-F = 400 µm.

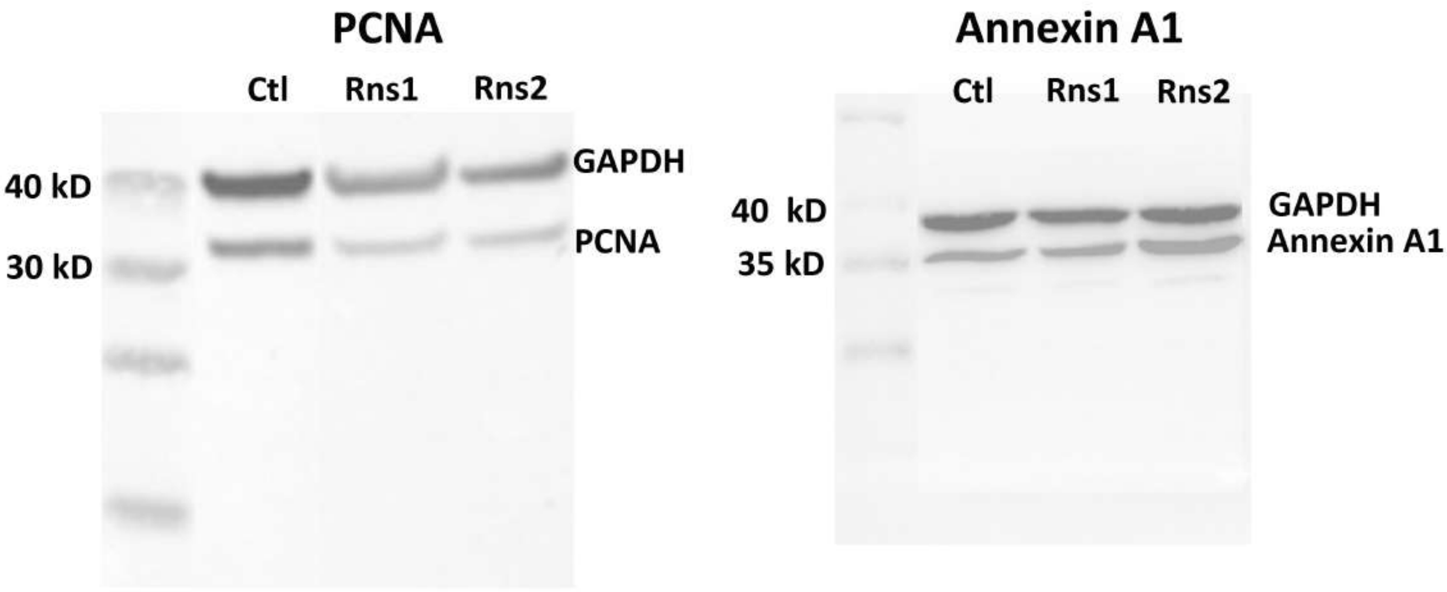

Supplementary Figure 3: Western blots were performed on cell lysates. PCNA protein levels were unchanged in RNS GFs compared to controls GFs.

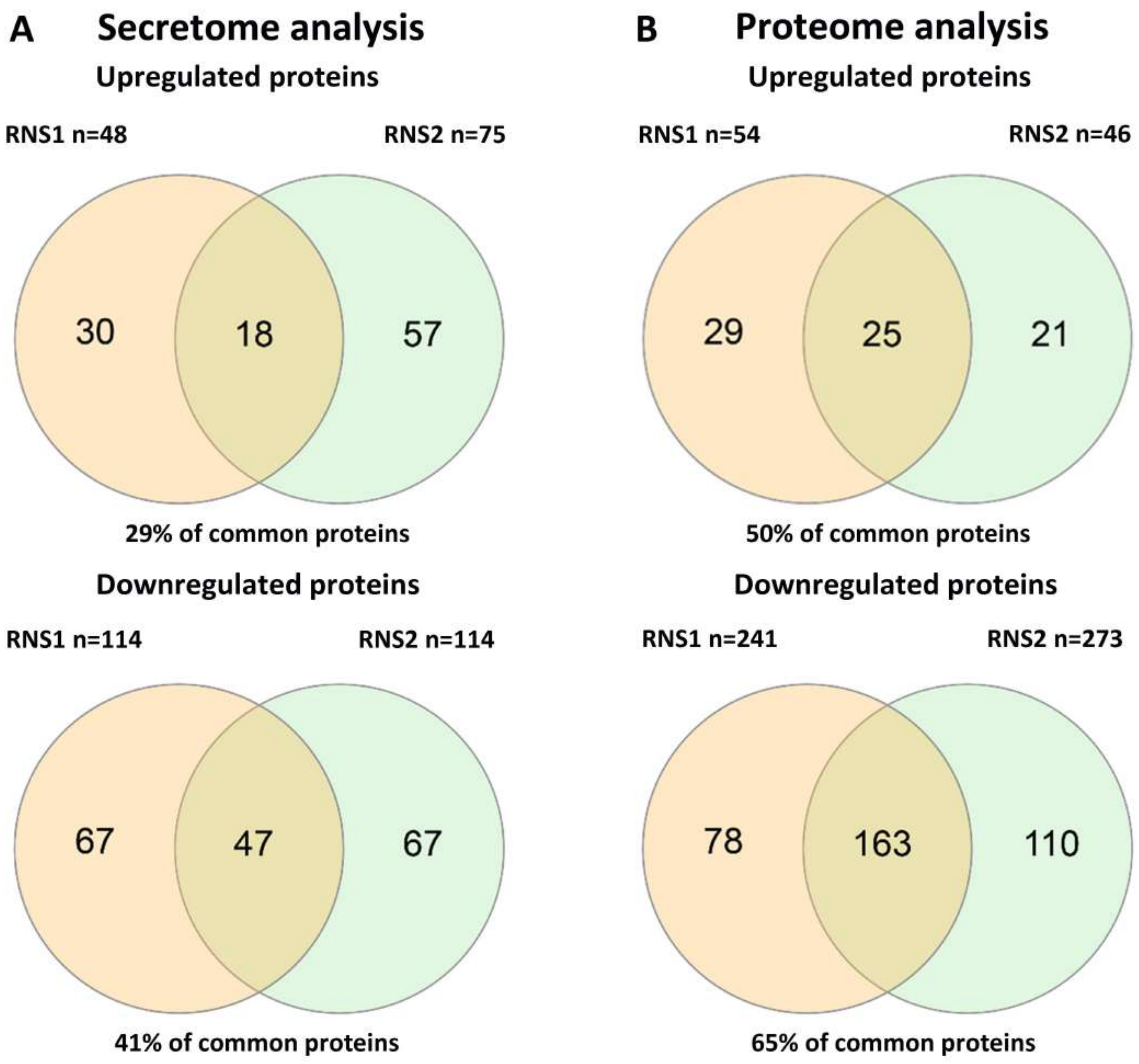

Supplementary Figure 4: (A) Venn diagram comparing the number of identified proteins in the secretome analysis between RNS-1 and RNS-2. The numbers of proteins detected with at least two different peptides are indicated by numbers. (B) Venn diagram comparing the number of identified proteins in the proteome analysis between RNS-1 and RNS-2. The numbers of proteins detected with at least two different peptides are indicated by numbers.

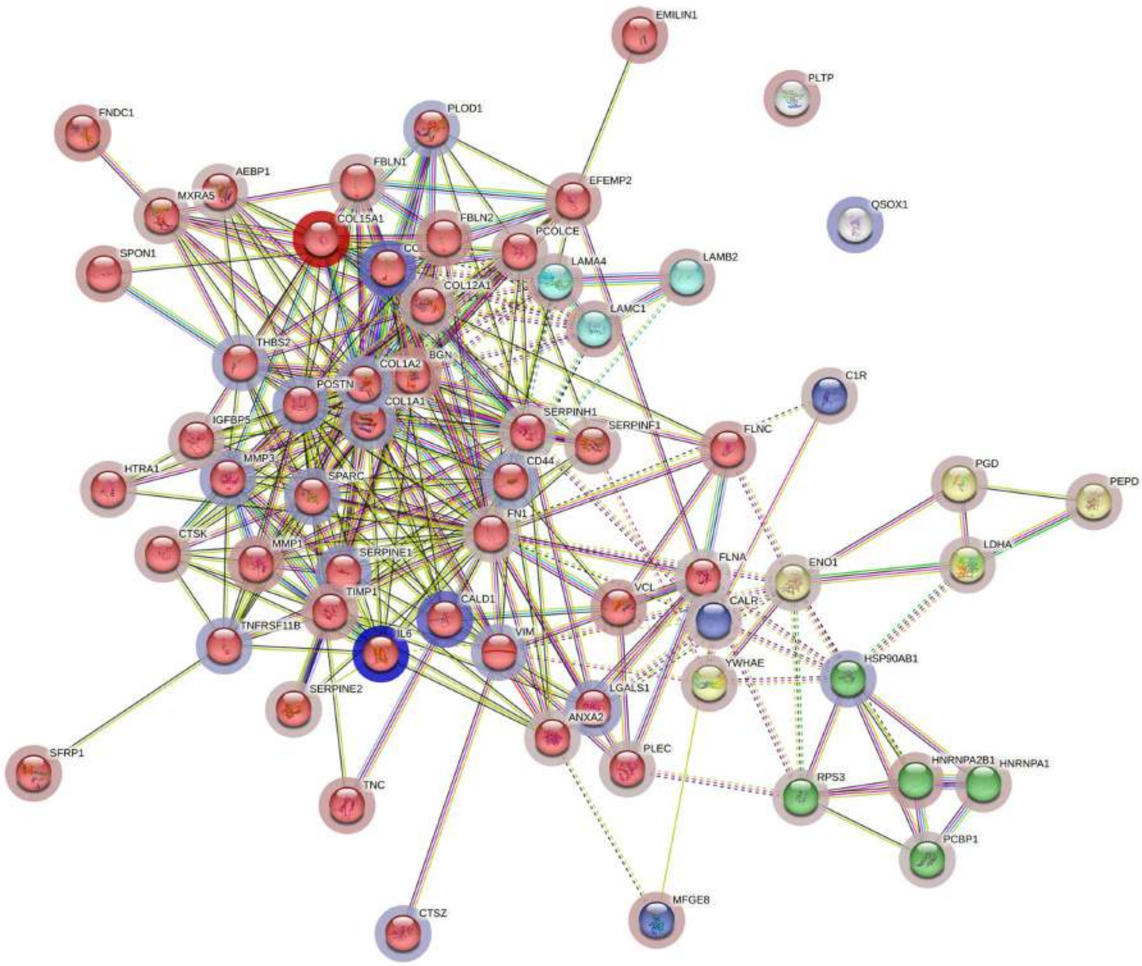

Supplementary Figure 5: Protein-protein association network using String analysis performed with the differentially secreted proteins, over-expressed and under-expressed in the RNS secretomes. Nodes in blue/grey circles stand for overrepresented proteins; Nodes in red/grey circles stand for underrepresented proteins. Code color clusterisation, protein names, identifications and descriptions are provided in Table S3.

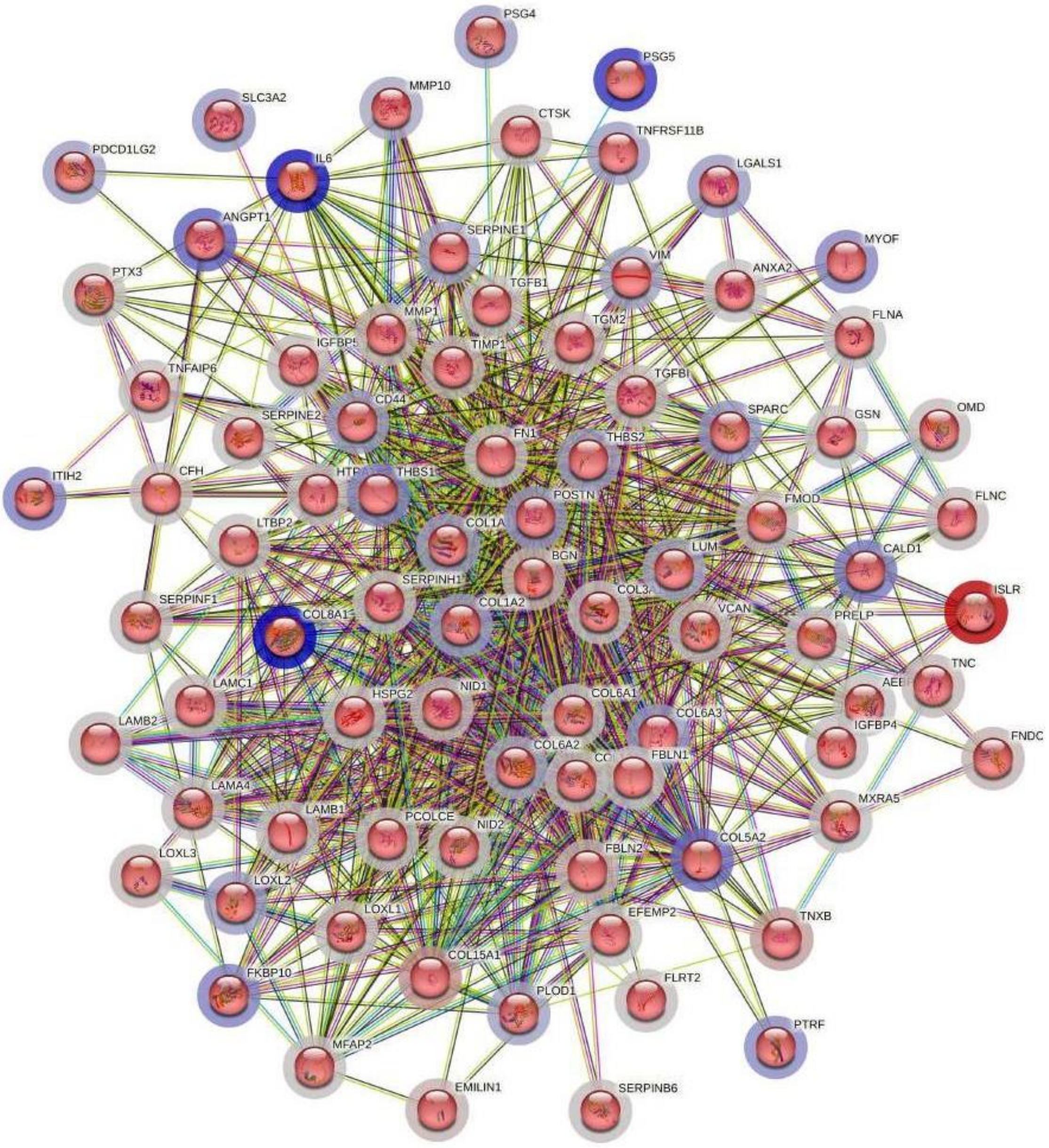

Supplementary Figure 6: Protein-protein association network using String analysis performed with all the differentially expressed proteins, over-expressed and under-expressed in the RNS-2 secretome. Nodes in blue/grey circles stand for overrepresented proteins; Nodes in red/grey circles stand for underrepresented proteins. Code color clusterisation, protein names, identifications and descriptions are provided in Table S6.

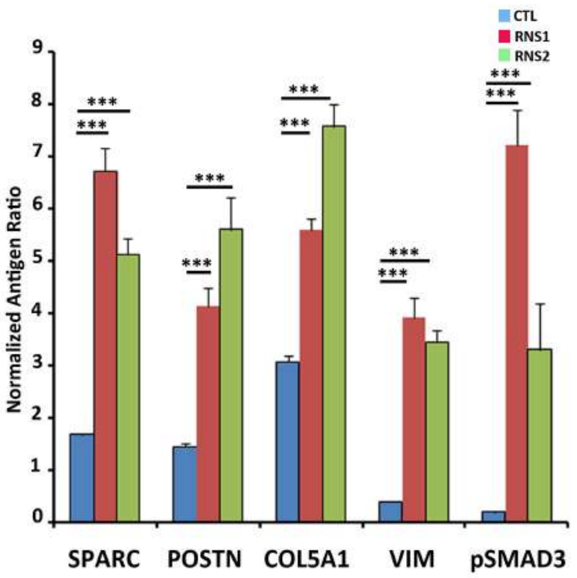

Supplementary Figure 7: Quantitative analysis of fluorescence staining on sections. Graph representation of the relative fluorescence intensity of SPARC, periostin (POSTN), alpha 1 collagen type 5 (COL5A1), vimentin (VIM), and phospho-SMAD3 in sections of control, RNS-1 and RNS-2 gingiva. Statistically significant differences are shown as ***P<0.001.

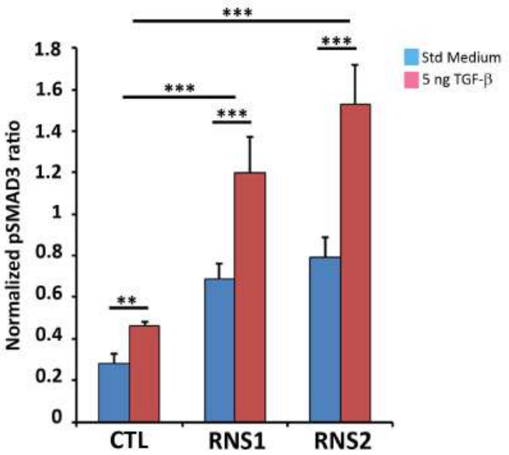

Supplementary Figure 8: Quantitative analysis of fluorescence staining. Graph representation of the relative fluorescence intensity of phospho-SMAD3 in gingival fibroblasts from control, RNS-1 and RNS-2, untreated or treated with 5ng/ml of TGF-beta. Statistically significant differences are shown as ***P<0.001.

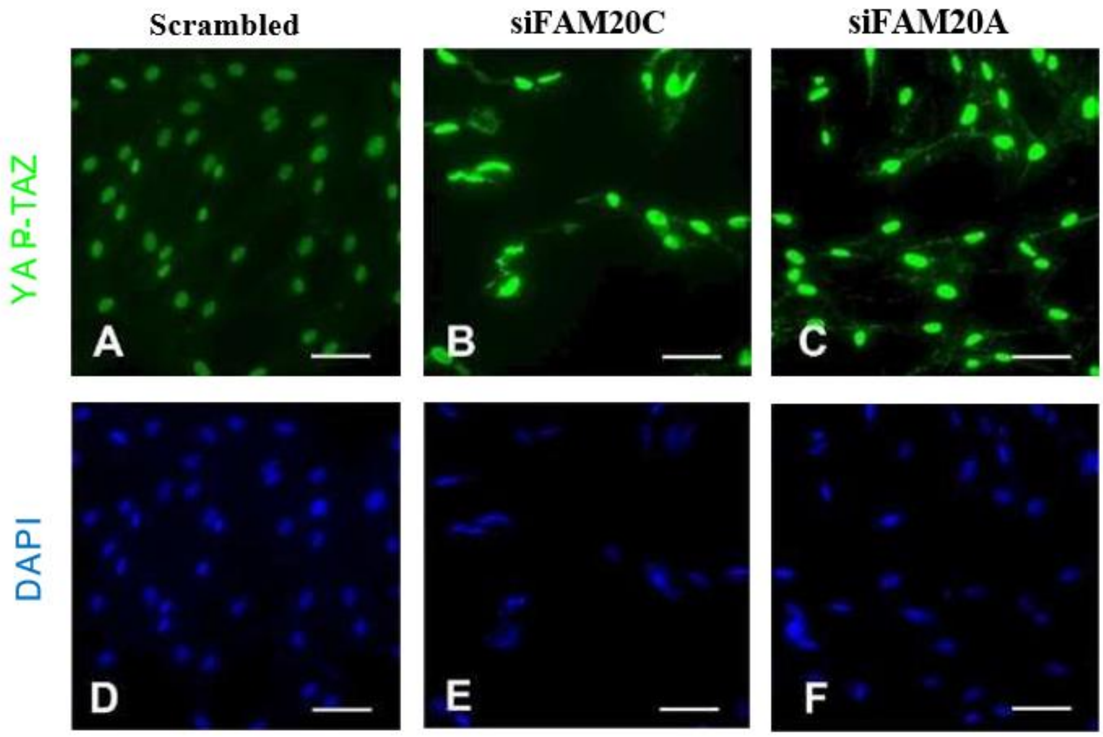

Supplementary Figure 9: Gingival fibroblasts were transfected with siRNAs targeted either scrambled siRNAs used as controls (A, D), *FAM20C* (B. E), or FAM*20A* (C, F). Three days after transfection, cells were analyzed for YAP-TAZ expression using immunocytochemistry (A-C). The cell nuclei were stained with DAPI (D-F). Scale bars = 50 µm.

8J

Yap397 No TGFB

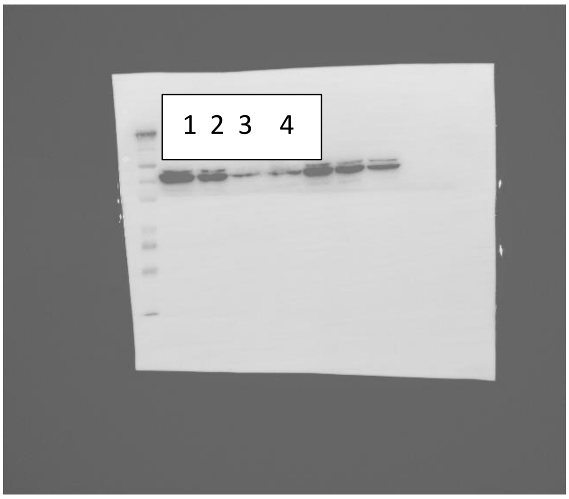

GAPDH of yap397-TGFB

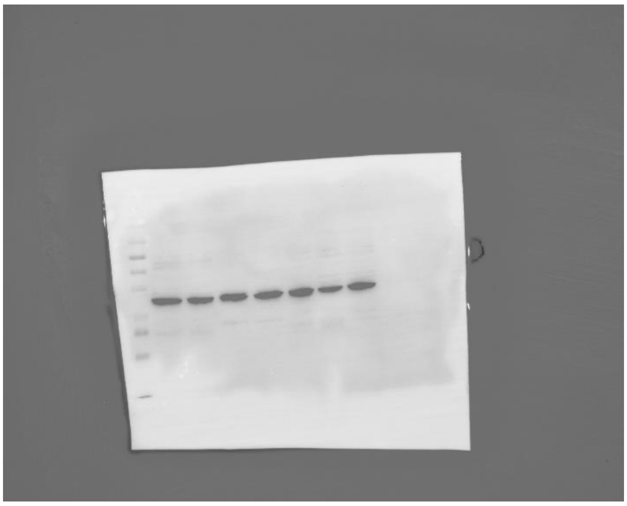

Yap397 +TGFB 5ng

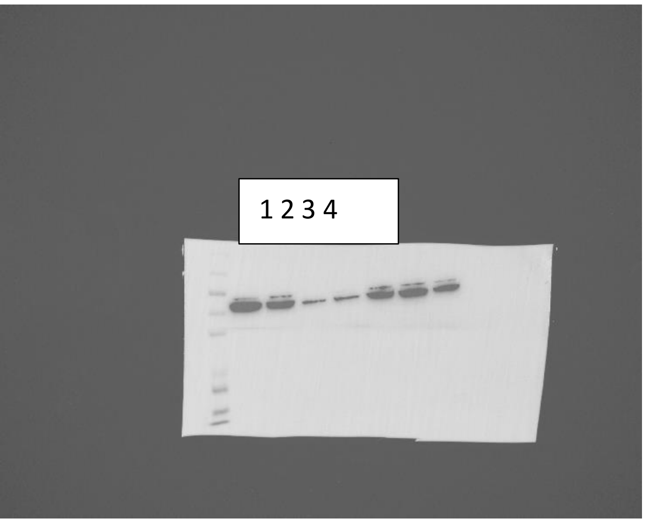

GAPDH of yap397+TGFB 5ng

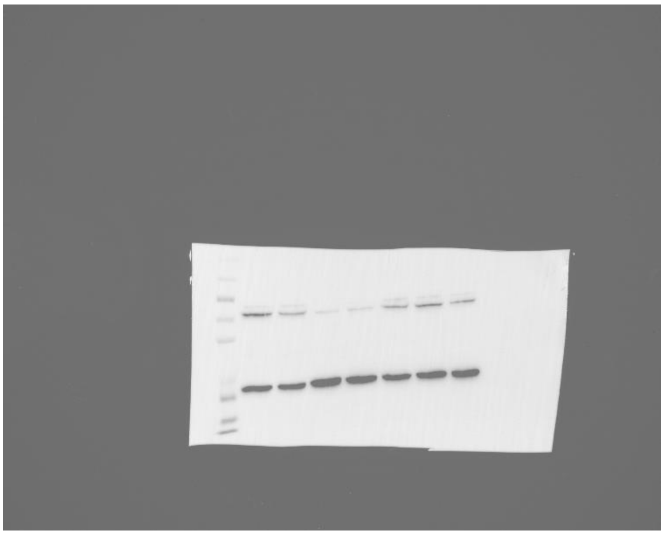

Yap taz NoTGFB

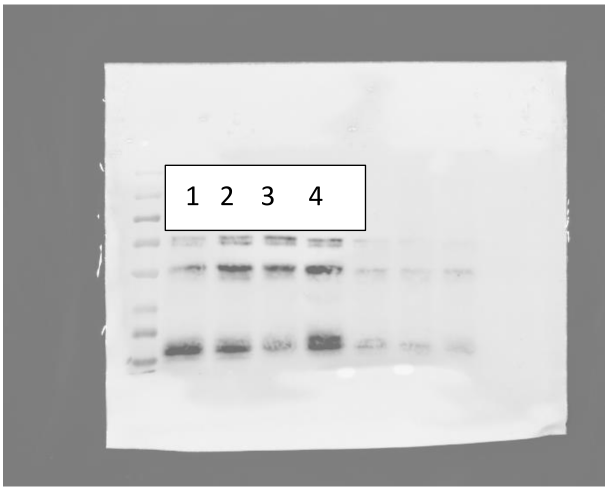

GAPDH of yap taz-TGFB

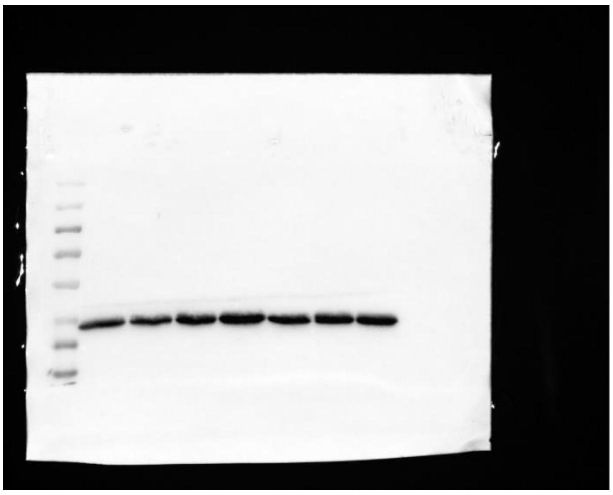

Yap taz +TGFB 5ng

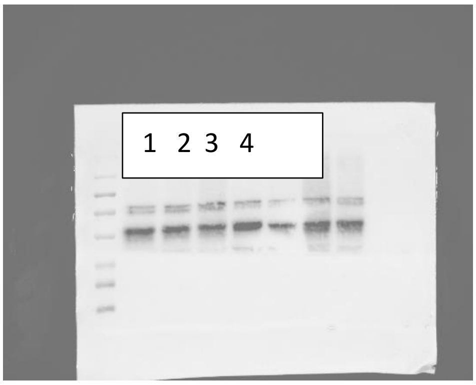

GAPDH of yap taz+TGFB 5ng

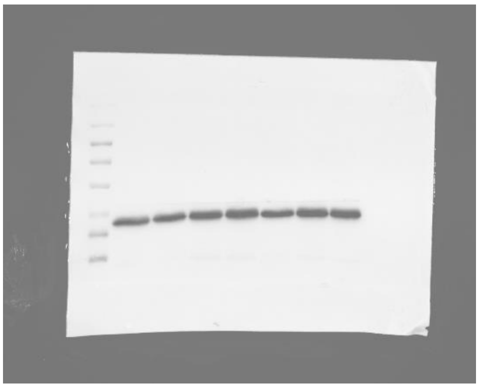

8H

Alpha sma No TGFB

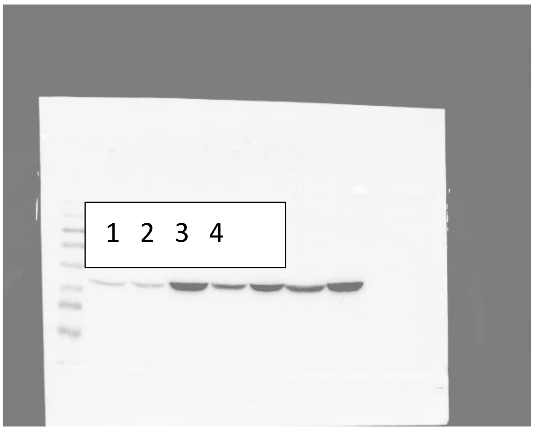

GAPDH of alpha sma -TGFB

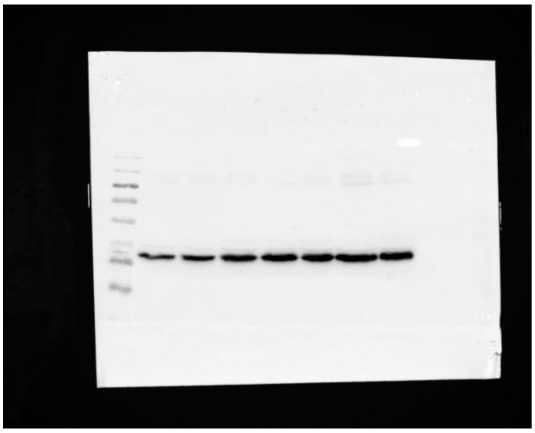

Alpha sma +TGFB 5ng

GAPDH ofAlpha sma +TGFB 5ng

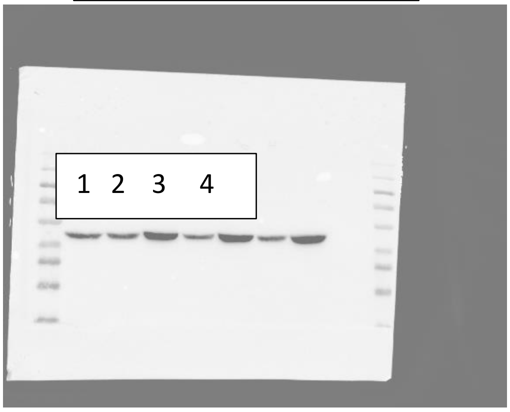

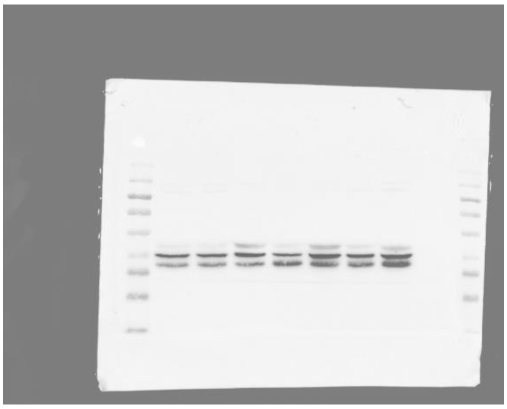

Annexine A1

GAPDH OF Annexine A1

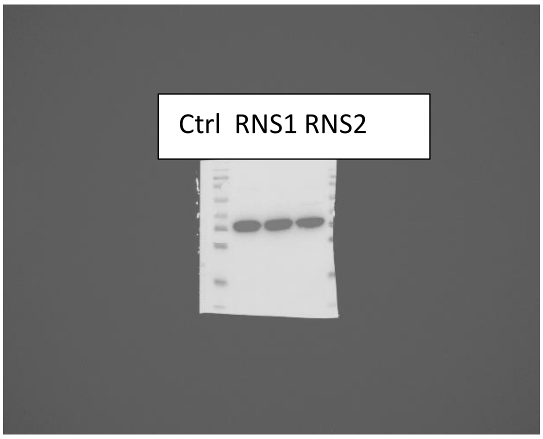

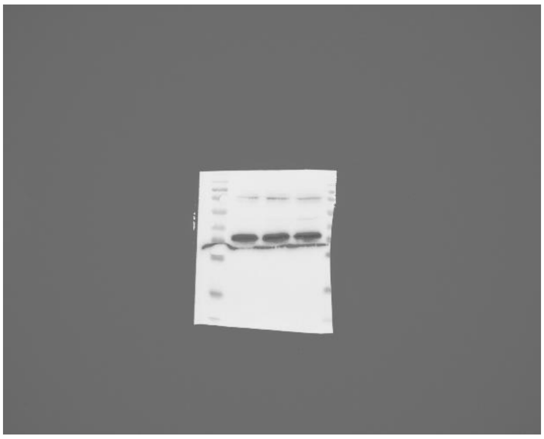

Supplementary fig3

Annexine A5

GAPDH of Annexine A5

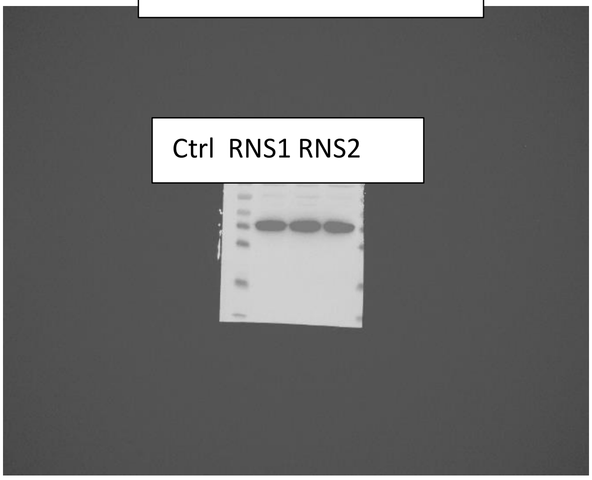

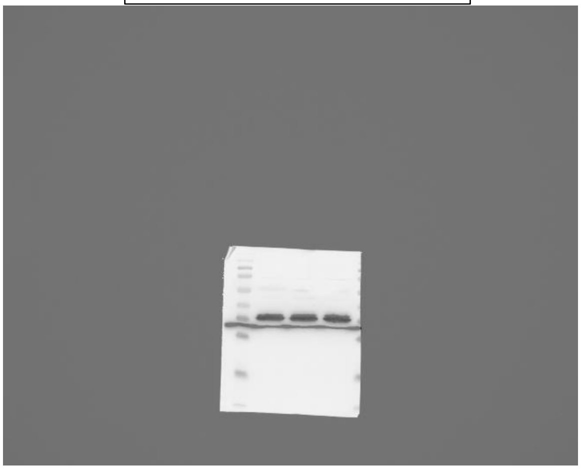

PCNA+GAPDH

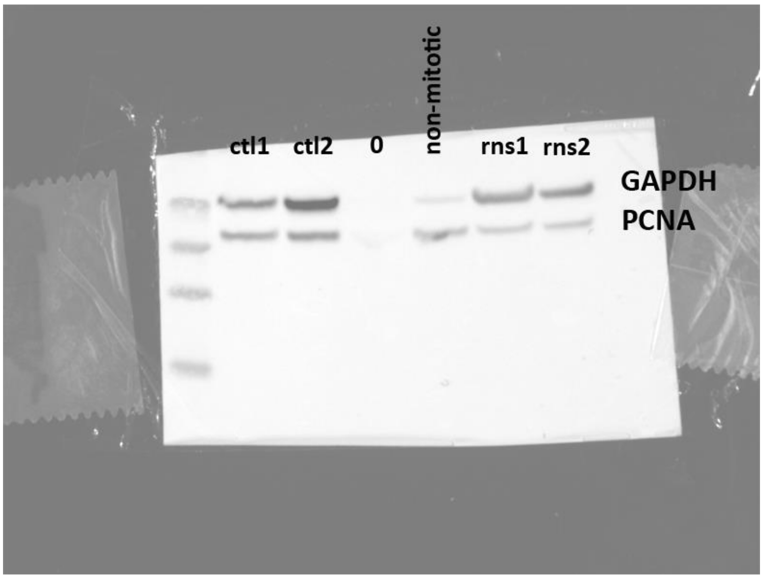

Unless otherwise indicated: Lanes 1, 2 are controls, Lane 3 is RNS1, Lane 4 is RNS2

Supplementary Figure 10: Original western blots for figure 8H,8J, PCNA, Annexin A1 and GAPDH (loading control).
