# Supplementary material for: Gingival proteomics reveals the role of TGF beta and YAP/TAZ signaling in Raine syndrome fibrosis
Source: Sci Rep. 2024 Apr 25;14:9497. doi: 10.1038/s41598-024-59713-0 (PMC11045870; doi:10.1038/s41598-024-59713-0)
Supplement: Supplementary file 2 — Supplementary Legends. [file 41598_2024_59713_MOESM2_ESM.docx]

**Supplementary material**

**Gingival proteomics reveals the role of TGF beta and YAP/TAZ signaling in Raine syndrome fibrosis**

Cláudio Rodrigues Rezende Costa^1,2,3^†, Rym Chalgoumi^1^†, Amina Baker^1^†, Clément Guillou^4,5^†, Paulo Marcio Yamaguti^2^, Victor Simancas Escorcia^1,6^, Lilia Abbad^7^, Bruna Rabelo Amorin^2^, Caroline Lourenço de Lima^2^, Vidjea Cannaya^1^, Mourad Benassarou^8^, Ariane Berdal^1,9^, Christos Chatziantoniou^6^, Olivier Cases^1^, Pascal Cosette^3,4^‡, Renata Kozyraki^1,9*^‡, Ana Carolina Acevedo^1,2^‡.

**14 Supplementary Tables in Excel Format:**

**Supplementary Table S1:** All proteins identified-secretome-controls-RNS1-RNS2

**Supplementary Table S2:** Differentially expressed proteins-secretome-CTLvsRNS

**Supplementary Table S3:** Common differentially expressed proteins-secretome-CTLvsRNS-analysis

**Supplementary Table S4:** MCL-clusters-RNS-Family1

**Supplementary Table S5:** Clusters-analysis-RNS-family1

**Supplementary Table S6:** MCL-clusters-RNS-Family2

**Supplementary Table S7:** Clusters-analysis-RNS-family2

**Supplementary Table S8:** All proteins identified-cell-lysate-controls-RNS1-RNS2

**Supplementary Table S9:** Differentially expressed proteins-cell-lysate

**Supplementary Table S10**: Common differentially expressed proteins-proteome-CTLvsRNS-analysis

**Supplementary Table S11:** Proteome-MCL-clusters-RNS-Family1

**Supplementary Table S12:** Proteome-clusters-analysis-RNS-family1

**Supplementary Table S13:** Proteome-MCL-clusters-RNS-Family2upplemenatry

**Supplementary Table S14:** Proteome-clusters-analysis-RNS-family2

**1 Supplementary Table in Word Format:**

**Supplementary Table S15:** List of primers used in the study

**8 Supplementary Figures**

**Supplementary Figure Legends:**

**Supplementary Figure 1:** (A) Immunohistochemical quantification of FAM20C and FAM20A on gingival sections from control (CTL), RNS-1 or RNS-2 specimens. Graph representation of the DAB quantification of FAM20C and FAM20A. Statistically significant differences are shown as ***P<0.001. (B) Quantitative analysis of fluorescence staining of FAM20C in gingival fibroblast *in vitro*. Graph representation of the relative fluorescence intensity of FAM20C in gingival fibroblasts from control, RNS-1 and RNS-2 cultures. Statistically significant differences are shown as ***P<0.001. (C, F, I) Immunostaining of FAM20C (red) and the nuclear specific marker, Lamin-B1 (green) in normal (C), RNS-1 (F) and RNS-2 (I) GFs. (D, G, J) Immunostaining of FAM20C (red) and the proteasome system marker, ubiquitin C-terminal hydrolase L1 (UCHL1, green) in normal (D), RNS-1 (G) and RNS-2 (J) GFs. (E, H, K) Immunostaining of FAM20C (red) and the lysosomal protein, Lamp1 (green) in normal (E), RNS-1 (H), and RNS-2 GFs. Scale bars: C, F, I, E, H, K = 40 μm; D, G, J = 25 μm.

**Supplementary Figure 2:** Histologic features of RNS mutants. (**A-C**) Hematoxylin-eosin staining shows numerous tortuous blood vessels (bv) with an abnormally large diameter in the papillary layer and associated extensive inflammatory infiltrates in RNS-1 gingiva (**B**). Abnormally large and shredded collagen bundles running in all directions are found in RNS-2 gingiva (**C**). (**D-F**) Intense Collagen I immunostaining in the connective tissue of RNS-1 and RNS-2 mutant gingivas. (**D**) Fibers of collagen organize in bundles of fixed diameter running in perpendicular pathways. In RNS-1 (**E**) and RNS-2 (**F**), fibers did not organize in packed bundles, but appeared shredded. Scale bars: A-C = 200 µm; D-F = 400 µm.

**Supplementary Figure 3**: Western blots were performed on cell lysates; detection of PCNA, Annexin A1and GAPDH (loading control) protein levels in RNS- and control GFs.

**Supplementary Figure 4**: Venn diagram analysis **(A)** Comparison of proteins up-regulated (upper part) and down-regulated (bottom part) between RNS1 and RNS2 in the cellular proteome analysis. For each comparison, the percentage of common proteins are mentioned. **(B)** Comparison of proteins up-regulated (upper part) and down-regulated (bottom part) between RNS1 and RNS2 in the secretome analysis. For each comparison, the percentage of common proteins are mentioned.

**Supplementary Figure 5**: Protein-protein association network using String analysis performed with the differentially secreted proteins, over-expressed and under-expressed in the RNS secretomes. Nodes in blue/grey circles stand for overrepresented proteins; Nodes in red/grey circles stand for underrepresented proteins. Code color clusterisation, protein names, identifications and descriptions are provided in Table S3.

**Supplementary Figure 6**: Quantitative analysis of fluorescence staining on sections. Graph representation of the relative fluorescence intensity of SPARC, periostin (POSTN), alpha 1 collagen type 5 (COL5A1), vimentin (VIM), and phospho-SMAD3 in sections of control, RNS-1 and RNS-2 gingiva. Statistically significant differences are shown as ***P<0.001.

**Supplementary Figure 7**: Quantitative analysis of fluorescence staining. Graph representation of the relative fluorescence intensity of phospho-SMAD3 in gingival fibroblasts from control, RNS-1 and RNS-2, untreated or treated with 5ng/ml of TGF-beta. Statistically significant differences are shown as ***P<0.001.

**Supplementary** **Figure 8**: Gingival fibroblasts were transfected with siRNAs targeted either scrambled siRNAs used as controls (A, D), *FAM20C* (B, E), or *FAM20A* (C, F). Three days after transfection, cells were analyzed for YAP-TAZ expression using immunocytochemistry (A-C). The cell nuclei were stained with DAPI (D-F). Scale bars = 50 μm.

**Supplementary Figure 10**: Original western blots for figure 8H,8J, PCNA, Annexin A1 and GAPDH (loading control).
