# Supplementary material for: Gingival proteomics reveals the role of TGF beta and YAP/TAZ signaling in Raine syndrome fibrosis
Source: Sci Rep. 2024 Apr 25;14:9497. doi: 10.1038/s41598-024-59713-0 (PMC11045870; doi:10.1038/s41598-024-59713-0)
Supplement: Supplementary file 17 — Supplementary Table S15. [file 41598_2024_59713_MOESM17_ESM.docx]

**Supplementary Table 15**. List of primers used in the study

| Gene | Primers |
| --- | --- |
| ENAH | ACAGGTGTATGGTCTCAACTTTG  TGTGAGTTTTGTCTAGGCAATGT |
| FNDC1 | ACCCAAAGATGCTACCAGTAGA  AGCAGCACAAAGTACACTACC |
| FAM20A | ACCAAGGAAATCCTAGAGGTCAC  CAGCATACTCCGTCTTGCACA |
| FAM20C | GGCACAATGCGGAGATTGC  CAGAGCTTCTTGTCCCGTGT |
| IL-6 | ACTCACCTCTTCAGAACGAATTG  CCATCTTTGGAAGGTTCAGGTTG |
| MMP3 | CGGTTCCGCCTGTCTCAAG  CGCCAAAAGTGCCTGTCTT |
| POSTN | CTCATAGTCGTATCAGGGGTCG  ACACAGTCGTTTTCTGTCCAC |
| SPARC | TGAGGTATCTGTGGGAGCTAATC  CCTTGCCGTGTTTGCAGTG |
| TGFB1 | CTAATGGTGGAAACCCACAACG  TATCGCCAGGAATTGTTGCTG |
| TGFB2 | CCCCGGAGGTGATTTCCATC  GGGCGGCATGTCTATTTTGTAAA |
| TGFBR2 | GTAGCTCTGATGAGTGCAATGAC  CAGATATGGCAACTCCCAGTG |
| TWF1 | ACGTGGGTGTGGACACTAAG  GGGAATCCTCTTTGGCAAATCTT |
